# CDC50 Orthologues in Plasmodium falciparum Have Distinct Roles in Merozoite Egress and Trophozoite Maturation

**DOI:** 10.1128/mbio.01635-22

**Published:** 2022-07-12

**Authors:** Avnish Patel, Stephanie D. Nofal, Michael J. Blackman, David A. Baker

**Affiliations:** a Department of Infection Biology, London School of Hygiene & Tropical Medicine, London, United Kingdom; b Malaria Biochemistry Laboratory, The Francis Crick Institute, London, United Kingdom; NIAID/NIH

**Keywords:** CDC50, P4-ATPase, *Plasmodium falciparum*, malaria, signal transduction

## Abstract

In model organisms, type IV ATPases (P4-ATPases) require cell division control protein 50 (CDC50) chaperones for their phospholipid flipping activity. In the malaria parasite Plasmodium falciparum, guanylyl cyclase alpha (GCα) is an integral membrane protein that is essential for release (egress) of merozoites from their host erythrocytes. GCα is unusual in that it contains both a C-terminal cyclase domain and an N-terminal P4-ATPase domain of unknown function. We sought to investigate whether any of the three CDC50 orthologues (termed A, B, and C) encoded by P. falciparum are required for GCα function. Using gene tagging and conditional gene disruption, we demonstrate that CDC50B and CDC50C but not CDC50A are expressed in the clinically important asexual blood stages and that CDC50B is a binding partner of GCα whereas CDC50C is the binding partner of another putative P4-ATPase, phospholipid-transporting ATPase 2 (ATP2). Our findings indicate that CDC50B has no essential role for intraerythrocytic parasite maturation but modulates the rate of parasite egress by interacting with GCα for optimal cGMP synthesis. In contrast, CDC50C is essential for blood stage trophozoite maturation. Additionally, we find that the CDC50C-ATP2 complex may influence parasite endocytosis of host cell hemoglobin and consequently hemozoin formation.

## INTRODUCTION

Plasmodium falciparum is responsible for the majority of malaria mortality and morbidity globally. While there was a sharp reduction in malaria-related deaths between 2000 and 2014 due to increased surveillance, improved control measures, and the use of highly effective drug treatments, the decline in cases has halted in recent years. This is thought to be due to the emergence of resistance to insecticides in the *Anopheles* mosquito vector and to the appearance of parasites resistant to artemisinin combination therapies (ACTs) (1). Given this trend, novel targets must be explored to generate candidates for the drug development pipeline to prevent a future increase in disease burden should the ACTs fail (2).

P. falciparum has a complex life cycle, characterized by multiple specialized developmental forms which transition between the mosquito vector and humans (3). Malaria pathology is caused exclusively by the asexual blood stage of the life cycle. Briefly, extracellular merozoites invade host erythrocytes (RBC) and transform rapidly into ring stages. Over the next ~24 h, these develop within a membrane-enclosed parasitophorous vacuole to form trophozoites which digest host cell hemoglobin and initiate DNA replication and endomitosis. The resulting schizonts undergo cytokinesis (segmentation) only upon maturation, forming daughter merozoites which are eventually released from the host cell ~48 h following invasion through a highly regulated egress process. The cycle is then reinitiated by the invasion of fresh erythrocytes by the newly released merozoites. A detailed molecular understanding of the biochemical pathways and proteins essential for blood stage development will inform discovery of novel targeted therapeutics that prevent malaria pathogenesis.

Merozoite egress and invasion are regulated by cyclic nucleotide signaling, conserved elements of which regulate multiple aspects of cell biology in model organisms and across the animal kingdom and can be effectively targeted pharmacologically (4). The second messengers, cyclic AMP (cAMP) and cyclic GMP (cGMP), are produced by cyclase enzymes and activate their respective cyclic nucleotide-dependent effector protein kinases PKA and PKG in a concentration-dependent manner (5). The activated kinases phosphorylate downstream targets that carry out effector functions, while the cyclic nucleotide signals are then broken down by phosphodiesterases (PDEs) (6, 7). P. falciparum uses cyclic nucleotide signaling throughout its complex life cycle (7). Notably, cGMP signaling is required for egress of asexual blood stage merozoites (8, 9) but also egress of gametes (10) and liver stage parasites (11, 12) as well as for regulation of ookinete and sporozoite motility (12–14). In contrast, cAMP signaling has been shown to be required for sporozoite apical organelle secretion and invasion of hepatocytes (15), gametocyte deformability (16), and erythrocyte invasion (17).

P. falciparum has two guanylyl cyclases. While guanylyl cyclase beta (GCβ) is dispensable in blood stages (9), guanylyl cyclase alpha (GCα) synthesizes cGMP in mature blood stage schizonts, where it plays an essential role in activating PKG to trigger egress (18). Both of the malaria parasite GCs are large integral membrane proteins with 22 predicted transmembrane domains (TMDs), the C-terminal segment of which constitutes the paired C1 and C2 guanylyl cyclase catalytic domains. Uniquely for cyclase enzymes, *Plasmodium* GCs (along with apicomplexan and ciliate orthologues) also contain an N-terminal type IV P-type ATPase (P4-ATPase)-like domain (18–20). In other organisms, P4-ATPases transport phospholipids from the outer to the inner leaflet of a lipid bilayer, maintaining the lipid asymmetry required for numerous functions, including membrane remodeling and vesicle formation (21). Recent studies in the apicomplexan parasite *Toxoplasma* indicate that this domain is critical to the role of its single guanylyl cyclase (T. gondii GC [TgGC]) in lytic growth, where it is essential for host cell attachment, invasion, and motility-dependent egress of tachyzoites (22–25).

In model organisms, P4-ATPases require cell division control protein 50 (CDC50) chaperones for their phospholipid flipping activity (26, 27). CDC50 proteins are integral membrane proteins with two TMDs that interact with a TMD of their partner P4-ATPases (28, 29). The intervening loop between the CDC50 TMDs forms a beta-sheet-rich structure that contacts the luminal side of P4-ATPases (28, 29). Studies in Saccharomyces cerevisiae have shown that CDC50 binding partners are required for the autophosphorylation of the catalytically active aspartic acid residue of the P4-ATPase, which is necessary for completion of the phospholipid flipping reaction cycle (30, 31). P. falciparum encodes three putative CDC50 proteins, termed CDC50A (PF3D7_0719500), CDC50B (PF3D7_1133300), and CDC50C (PF3D7_1029400). Previous work in the mouse malaria model Plasmodium yoelii has shown that the CDC50A orthologue binds to GCβ and is required for ookinete motility (20). Similarly, TgGC controls egress of tachyzoites (22, 24, 25) and binds to a *Toxoplasma* CDC50 partner that is required for its function (22). However, the functions of P. falciparum CDC50 orthologues have not been examined. Here, we show that both CDC50B and CDC50C, but not CDC50A, are expressed in the asexual blood stages and that CDC50B interacts with GCα whereas CDC50C is the binding partner of another putative P4-ATPase (ATPase2; PF3D7_1219600). We show that CDC50B modulates the efficiency of parasite egress by interacting with GCα for optimal cGMP synthesis. In contrast, CDC50C is essential for asexual blood stage trophozoite maturation due to a crucial role in endocytosis of host erythrocyte hemoglobin.

## RESULTS

### Generation of genetic tools to investigate the function of the P. falciparum CDC50s.

**FIG 1:**
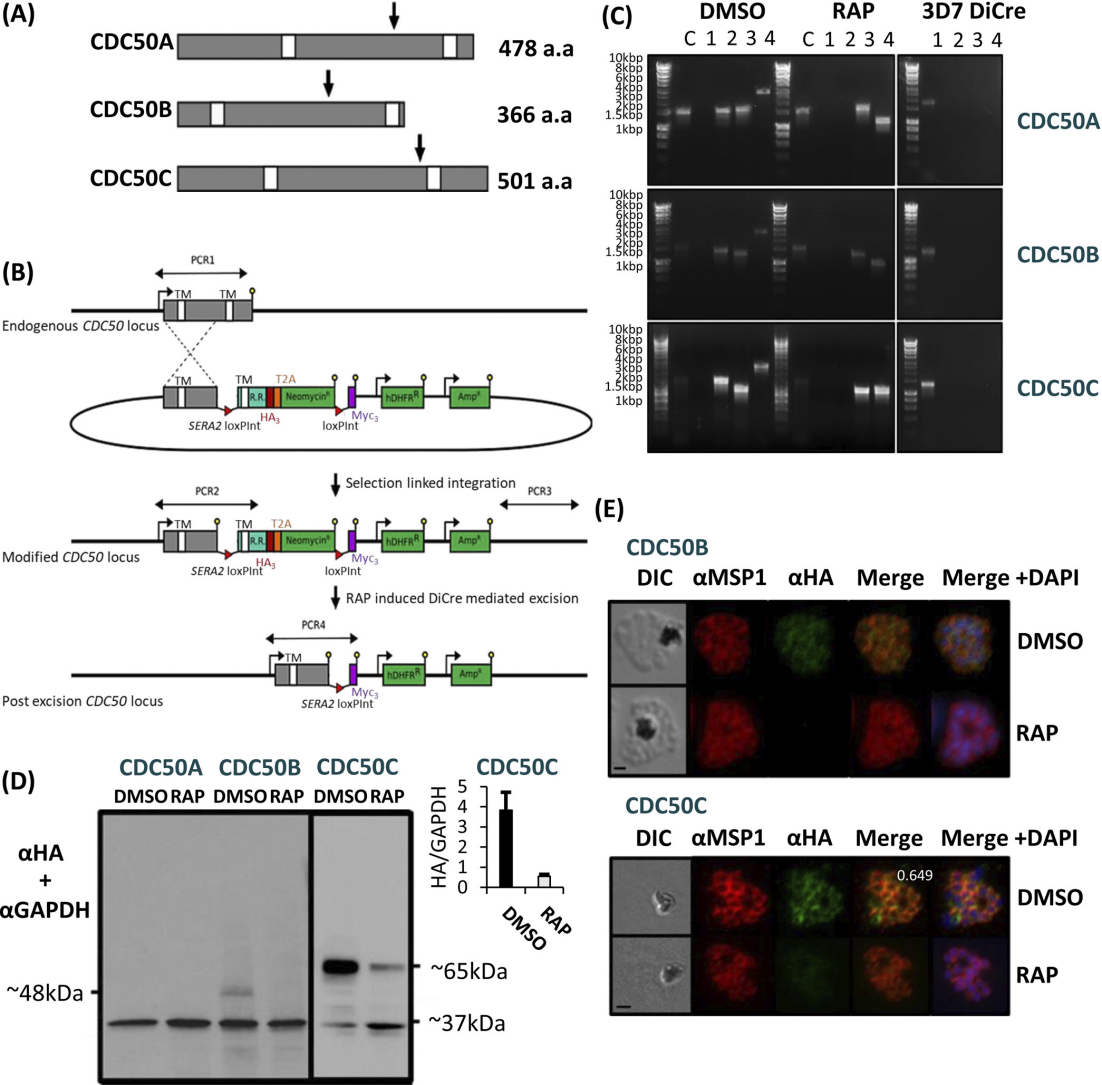
(A) Representation of the three P. falciparum CDC50 proteins displayed from the N terminus to the C terminus. White boxes, transmembrane helices (TMDs) as predicted by TMHMM (59). Arrows indicate the point from which the protein products are truncated when the corresponding modified locus is excised in transgenically modified parasite lines. For CDC50A, this is from Phe341, for CDC50B from His235, and for CDC50C from Glu383. a.a, amino acids. (B) Schematic representation of the SLI strategy (58) used to produce the three CDC50 DiCre lines and resultant RAP-induced disruption of the modified genes. Double-headed arrows, regions amplified by PCR in panel C; red arrowheads, loxP sites; yellow lollipops, translational stop codons; white boxes, TMDs; light blue boxes, recodonized sequence (R.R.). (C) Diagnostic PCR analysis of gDNA from transgenic CDC50 parasite lines CDC50A-HA:loxP, CDC50B-HA:loxP, and CDC50C-HA:loxP verifying successful modification of target loci by SLI. Efficient excision of “floxed” sequences is observed in RAP treatment for all lines. Lane C represents amplification of a control locus (PKAc) to check gDNA integrity. PCRs 1 to 4 are represented in the schematic locus in panel B. PCR 1 screens for the WT locus, PCR 2 for 5′ integration, PCR 3 for 3′ integration, and PCR 4 for excision of the floxed sequence. See Table 1 for sequences of all primers used for PCR. Sizes of expected amplicons are as follows. C, control locus (primers 16 and 17), 1,642 bp. For CDC50A: PCR 1 (primers 21 and 22), 1,842 bp; PCR2 (primers 21 and 18), 1,613 bp; PCR3 (primers 20 and 22), 1,670 bp; and PCR 4 (primers 21 and 19), 2,863 bp (DMSO) and 1,169 bp (RAP). For CDC50B: PCR 1 (primers 23 and 24), 1,423 bp; PCR2 (primers 23 and 18), 1,457 bp; PCR3 (primers 20 and 24), 1,321 bp; and PCR 4 (primers 23 and 19), 2,707 bp (DMSO) and 1,010 bp (RAP). For CDC50C: PCR 1 (primers 25 and 26), 1,369 bp; PCR2 (primers 25 and 18), 1,602 bp; PCR3 (primers 20 and 26), 1,172 bp; and PCR 4 (primers 25 and 19), 2,852 bp (DMSO) and 1,369 bp (RAP). (D) Western blot analysis of expression (DMSO) and ablation (RAP) of CDC50A-HA, CDC50B-HA, and CDC50C-HA from highly synchronous late-stage schizonts in the respective transgenic parasite lines. Expression of GAPDH (PF3D7_1462800) is shown as a loading control. CDC50C-HA showed some residual expression after excision, but quantification shows an ~8-fold reduction in protein following RAP treatment (inset). No expression of CDC50A-HA was detected. Predicted molecular masses of CDC50B-HA, CDC50C-HA, and GAPDH are indicated. (E) IFA analysis showing diffuse peripheral localization of CDC50B-HA and CDCD50C-HA and loss of expression upon RAP treatment (16 h postinvasion). Over 99% of all RAP-treated CDC50B-HA:loxP and CDC50C-HA:loxP schizonts examined by IFA were diminished in HA expression in three independent experiments. Signals are representative of fields of view containing at least 10 parasites from three independent experiments. The inset number in the merge panel for DMSO represents the Pearson correlation coefficient for the HA and MSP1 signals. Scale bar, 2 μm.

To assess the biological functions of CDC50A, CDC50B, and CDC50C in P. falciparum blood stages, we generated three transgenic parasite lines designed to allow investigation of subcellular location and the effects of conditional disruption of each CDC50. The transgenics were generated in the genetic background of a 3D7 P. falciparum line that stably expresses dimerizable Cre (DiCre), the Cre recombinase activity of which is induced in the presence of rapamycin (RAP) (32, 33). In each case, the target genes were “floxed” such that treatment with RAP would lead to excision of DNA sequences encoding a C-terminal region containing the second TMD of each protein (Fig. 1A and B); this TMD has been shown in model organism CDC50-ATPase structures to interact with the most C-terminal helix of the ATPase binding partner (28, 29). The constructs were designed so that following homologous recombination, the genes were also modified by fusion to sequences encoding a C-terminal triple hemagglutinin (HA) epitope tag.

Successful modification of the target genes was verified by PCR (Fig. 1C), and expression and RAP-induced truncation of tagged CDC50A-HA, CDC50B-HA, and CDC50C-HA in the respective transgenic parasites (termed CDC50A-HA:loxP, CDC50C-HA:loxP, and CDC50B-HA:loxP) was confirmed by Western blotting (Fig. 1D). Immunofluorescence analysis (IFA) of the transgenic lines (Fig. 1E) revealed a diffuse, partly peripheral signal in individual merozoites within mature segmented schizonts for both CDC50B-HA and CDC50C-HA. This was similar to the pattern observed upon costaining with the plasma membrane marker merozoite surface protein 1 (MSP1). While successful tagging and floxing of the *CDC50A* gene was also confirmed by PCR and Sanger sequencing, no protein expression could be detected in asexual blood stages. This suggested that CDC50A is not expressed in asexual stages, consistent with findings in P. yoelii, where CDC50A is expressed only in gametocyte and mosquito stages (20). Alternatively, since P. falciparum transcriptomic data indicate that the *CDC50A* gene is transcribed in schizont stages (34), the protein may be expressed but rapidly degraded as its GCβ binding partner is not present in asexual blood stages (9, 19).

### Of the three isoforms, only CDC50C is essential for blood stage parasite growth.

**FIG 2:**
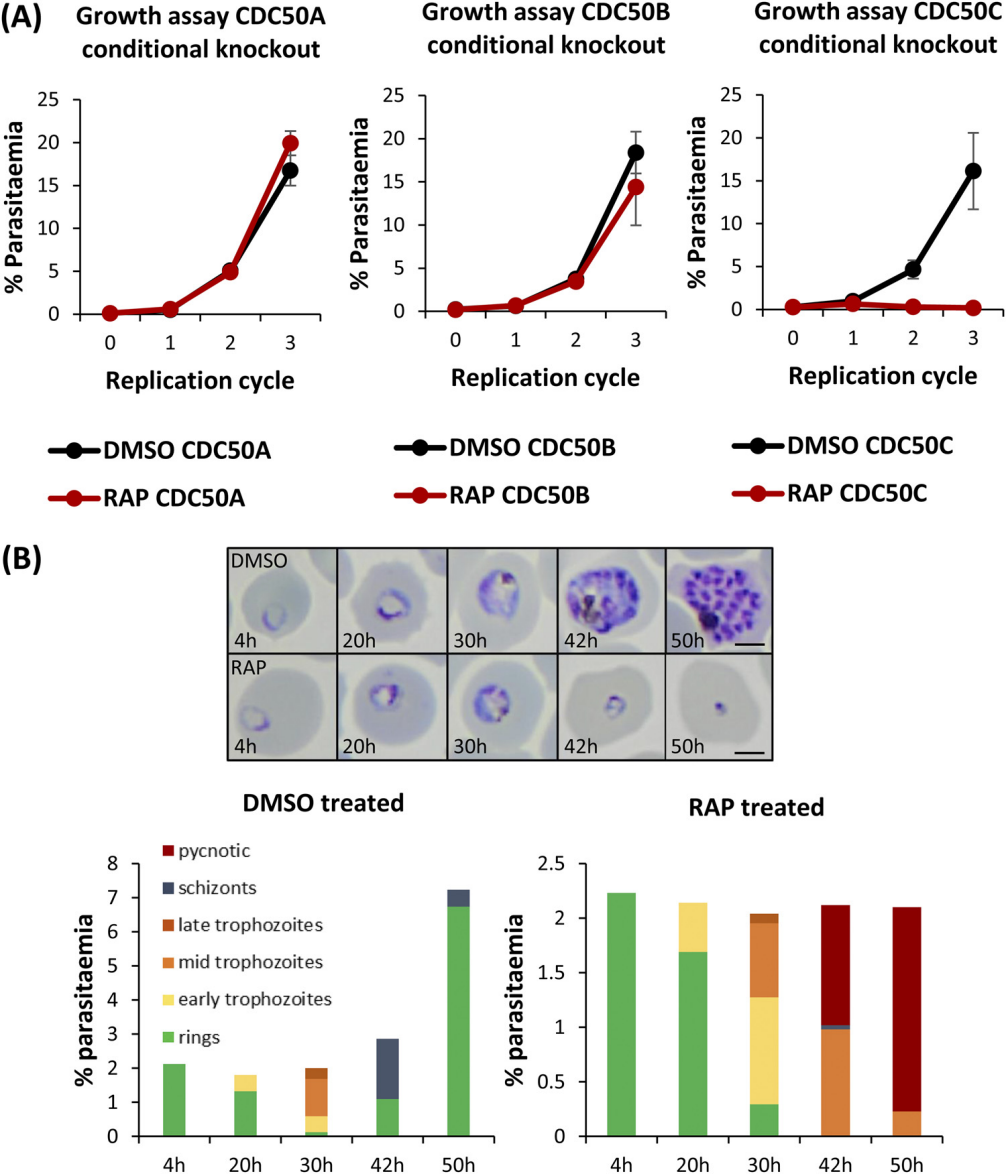
(A) Growth curves showing parasitemia as measured by flow cytometry of CDC50A-HA:loxP, CDC50B-HA:loxP, and CDC50C-HA:loxP parasites treated with DMSO (vehicle-only control) or RAP. Means of results from three independent experiments are plotted. Error bars, standard deviations (SD). (B) Upper panel, Giemsa-stained thin blood films showing development of ring stage parasites following egress of synchronous DMSO- and RAP-treated CDC50C-HA:loxP schizonts. Ring formation occurs in RAP-treated CDC50C-HA parasites, but the parasites did not develop beyond the early trophozoite stage and eventually collapsed into small vacuoles. Scale bars, 2 μm. Lower panel, microscopic quantification of parasite developmental stages at each time point. RAP-treated parasites displayed an accumulation of pycnotic parasites at late life cycle stages and showed no expansion in parasitemia. Counts are means of results of two independent experiments.

To investigate the essentiality of CDC50A, CDC50B, and CDC50C, highly synchronized ring stage cultures of each DiCre transgenic line were treated with RAP to induce excision of the sequence encoding the C-terminal TMD of each protein (Fig. 1C), and parasite replication was assessed using flow cytometry. RAP-treated CDC50A-HA:loxP and CDC50B-HA:loxP parasites displayed no significant growth inhibition over three cycles compared to matched control (dimethyl sulfoxide [DMSO]-treated) parasites (Fig. 2A). In contrast, CDC50C-HA:loxP parasites underwent complete growth arrest after cycle 1. Examination of the parasites by Giemsa staining showed that while new rings went on to form schizonts in DMSO-treated wild-type (WT) parasites, RAP-treated CDC50C-HA:loxP rings did not develop beyond the early trophozoite stage and displayed an accumulation of pycnotic parasites 30 h postinvasion with no expansion in parasitemia (Fig. 2B). It was concluded that CDC50C is essential for asexual blood stage survival.

### CDC50B and CDC50C bind to distinct parasite flippase partners.

**FIG 3:**
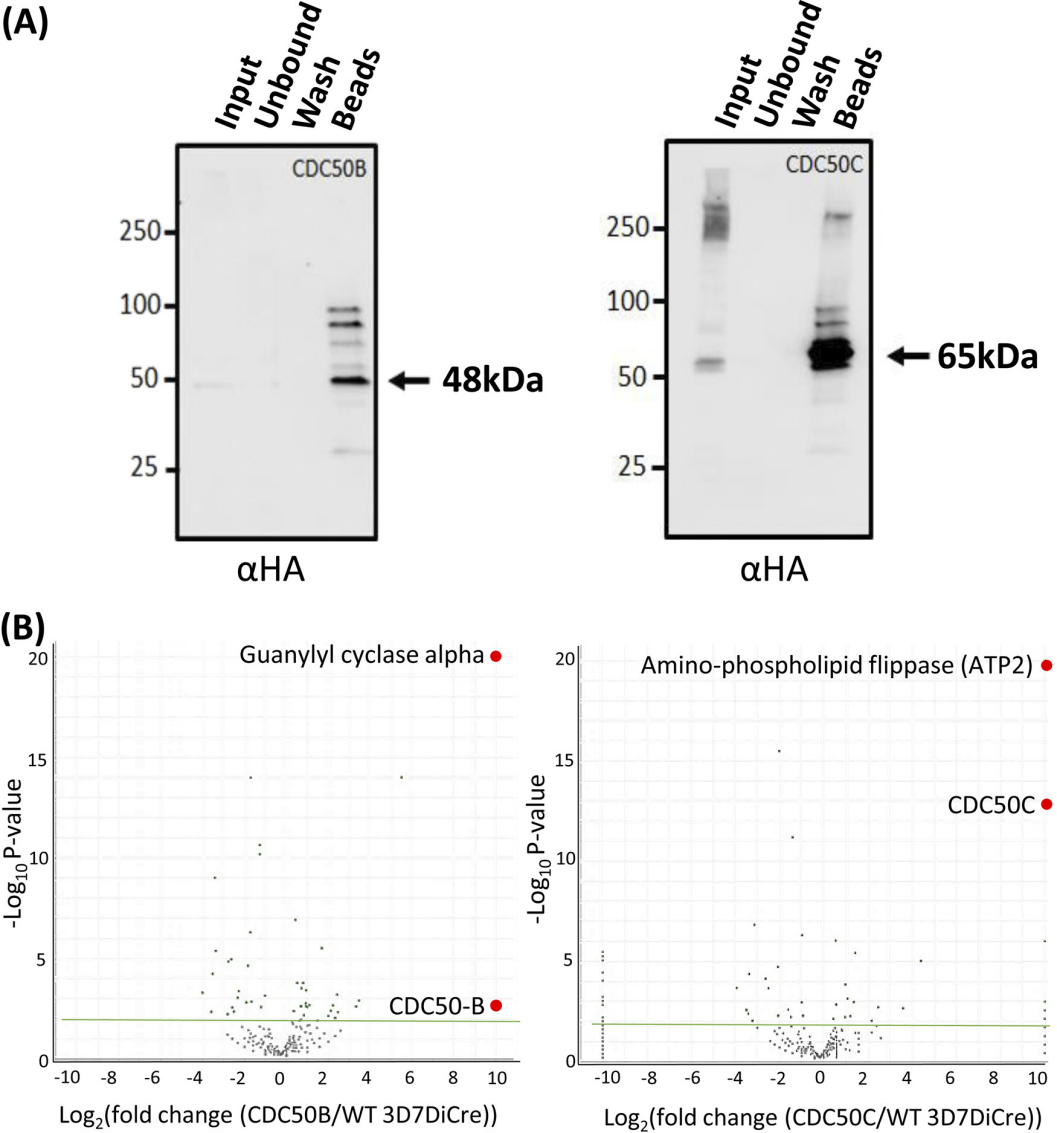
(A) Western blotting demonstrates efficient immunoprecipitation (IP) of CDC50B-HA and CDC50C-HA from schizont extracts. Arrows indicate the predicted mass of each protein. Images are representative of two independent experiments. (B) Mass spectrometric identification of interacting partners of CDC50B and CDC50C by analysis of proteins enriched through IP (panel A). Volcano plot of *P* values versus the corresponding log_2_ fold change in abundance compared to 3D7DiCre control samples (Fisher’s exact test). A green line indicates a *P* of −2log_10_, and green dots represent peptides where the *P* is less than −2log_10_. Peptides for GCα and ATP2 were enriched to a *P* of less than −19log_10_. A list of enriched proteins is found in Table S1 in the supplemental material.

In other organisms, CDC50 proteins interact with their cognate P4-ATPases and are required for their activity (28–31). To determine whether CDC50B and CDC50C interact with P4-ATPases during P. falciparum blood stage development, we performed immunoprecipitation (IP) experiments from extracts of highly synchronized CDC50B-HA:loxP and CDC50C-HA:loxP schizonts. Western blot analysis confirmed the expected enrichment of the HA-tagged proteins from schizont lysates (Fig. 3A). The immunoprecipitated material was then analyzed by mass spectrometry in comparison with mock IP samples derived from 3D7DiCre parental parasites, to confirm this and to identify coprecipitating protein species. As shown in Fig. 3B, this confirmed high levels of enrichment of the HA-tagged CDC50 bait proteins. In addition, in the case of the CDC50B experiments, we detected a >9 log_2_ enrichment of peptides derived from GCα (Fig. 3B), while in the CDC50C IPs, we detected >9 log_2_ enrichment of peptides mapping to another putative P4-ATPase, a putative aminophospholipid flippase (PF3D7_1219600) (ATP2) (Fig. 3B). No other proteins were as significantly enriched in each pulldown. These results strongly suggest that CDC50B is a cofactor for GCα and that CDC50C is a cofactor for ATP2.

10.1128/mbio.01635-22.10 TABLE S1 List of enriched proteins. Download Table S1, XLSX file, 0.01 MB. Copyright © 2022 Patel et al. 2022 Patel et al. https://creativecommons.org/licenses/by/4.0/ This content is distributed under the terms of the Creative Commons Attribution 4.0 International license.

### CDC50B is not required for GCα expression or trafficking but is crucial for optimal cGMP synthesis required for egress.

GCα has a key role in egress as the source of cGMP required for PKG activation (18). Having determined that CDC50B interacts with GCα, we next investigated whether CDC50B also has a role in parasite egress.

**FIG 4:**
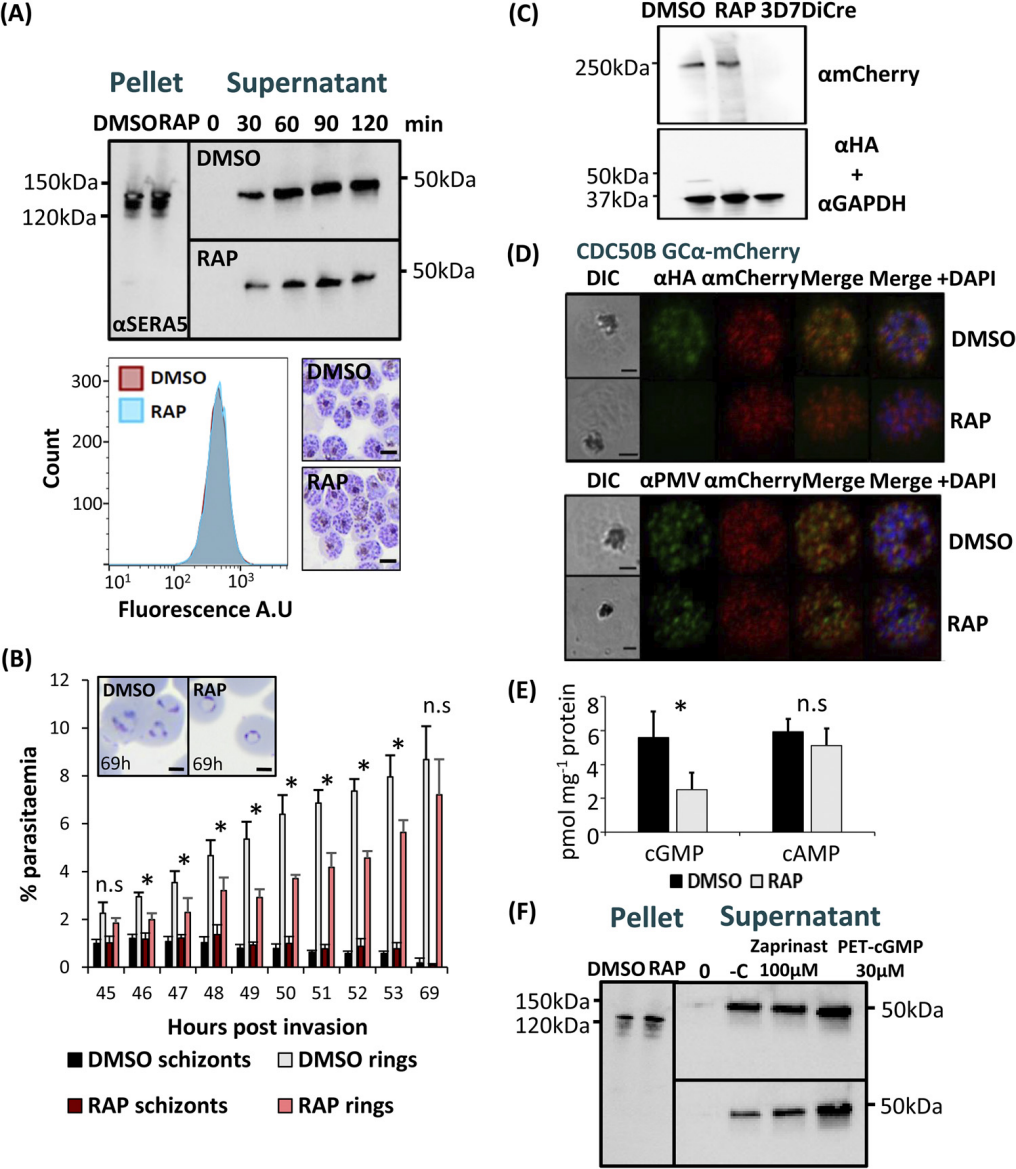
(A) Western blot analysis monitoring egress kinetics of DMSO- and RAP-treated CDC50B-HA:loxP schizonts. Reduced detection of the SERA5 p50 proteolytic fragment in culture supernatants of RAP-treated CDC50B-HA:loxP parasites indicates an impaired egress rate in the absence of CDC50B-HA. Lower panel left, histograms of DNA (SYBR green) staining of DMSO- or RAP-treated schizonts. A total of 10,000 cells were counted per treatment. The image is representative of three independent experiments. Lower panel right, Giemsa-stained thin blood films of Percoll-purified DMSO- and RAP-treated schizonts. No delay in schizont maturation is evident in RAP-treated parasites. Images are representative of three independent experiments. Scale bar, 5 μm. (B) Flow cytometry analysis of ring formation by DMSO- and RAP-treated CDC50B-HA:loxP parasites. Samples from highly synchronized cultures treated at the ring stage were taken in triplicate at hourly intervals from 45 to 53 h and at 69 h postinvasion were stained with the DNA stain SYBR green. Samples were analyzed by flow cytometry, and the schizont and ring parasitemias were determined by gating high-signal and low-signal SYBR-positive cells, respectively. Mean parasitemia values (starting schizontemia adjusted to 2%) from two independent experiments are plotted. Error bars, SD; n.s, not significant; *, *P* < 0.05, by Student's *t* test for comparison of ring parasitemias between DMSO and RAP samples. Insets are smears taken from cultures at 69 h postinvasion. Scale bars, 2 μm. (C) Western blot analysis of DMSO- and RAP-treated CDC50B-HA:loxP GCα-mCherry and control 3D7DiCre schizonts. Top panel, ~250-kDa fragment detected by an mCherry antibody that is absent from control (untagged) schizont lysates. Lower panel, the same samples probed with an anti-HA antibody and an anti-GAPDH (PF3D7_1462800) loading control antibody. (D) Top, localization by IFA of CDC50B and GCα mCherry in RAP- and DMSO-treated CDC50B-HA:loxP GCα-mCherry schizonts. Bottom, localization of GCα mCherry and PMV (PF3D7_1323500), an ER marker in RAP- and DMSO-treated schizonts. Scale bar, 2 μm. (E) Quantification of cyclic nucleotide levels in tightly synchronized DMSO- and RAP-treated mature CDC50B-HA:loxP schizonts by direct ELISA. Means of results from three independent experiments are plotted. Error bars, SD; n.s, not significant; *, *P* < 0.05, Student's *t* test. (F) Restoration of egress of RAP-treated CDC50B-HA:loxP schizonts by treatment with zaprinast or PET-cGMP. Supernatant and pellet samples were taken at time point 0 after washing with RPMI 1640 medium, to control for parasite numbers and egress. Samples were then taken at 60 min postincubation at 37°C. Lane −C, no treatment. The image is representative of three independent experiments.

To do this, we first compared the egress kinetics of mature DMSO- and RAP-treated CDC50B-HA:loxP schizonts by monitoring the appearance in culture supernatants over time of proteolytically processed forms of the PV protein serine repeat antigen 5 (SERA5), which acts as a proxy for egress due to its release upon schizont rupture (35). As shown in Fig. 4A, these experiments revealed a marked reduction in the rate of egress over the sampling period in RAP-treated CDC50B-HA:loxP parasites compared to that of control DMSO-treated counterparts. Densitometric quantitation of data from three independent experiments indicated that CDC50B null schizonts undergo ~50% less egress than WT controls when sampled over a 2-h period (see Fig. S1 in the supplemental material). This was not due to a delay in schizont development, since microscopic examination of Giemsa-stained DMSO- and RAP-treated CDC50B-HA:loxP schizonts showed no detectable delay in parasite maturation, and analysis of DNA content by flow cytometry indicated no significant differences between formation of DMSO- and RAP-treated CDC50B-HA:loxP schizonts (Fig. 4A, lower panel). We further investigated the egress phenotype of CDC50B null parasites using a flow cytometry time course to assess the production of new rings by highly synchronized DMSO- and RAP-treated CDC50B-HA:loxP schizonts. We observed that while the number of new rings formed in the CDC50B null population between 45 and 53 h postinvasion was reduced, by 69 h postinvasion there was no statistically significant difference in ring parasitemia between the control and CDC50B null parasites (Fig. 4B). Microscopic examination of these newly generated ring stage parasites showed that those derived from the DMSO-treated parasites appeared more mature than those derived from the RAP-treated cultures (Fig. 4B, inset boxes), suggesting a delay to invasion in the latter. Together, the data indicate that CDC50B null parasites exhibit an extended erythrocytic life cycle, resulting in a delay to egress after schizont maturation but with no overall reduction in new ring stage formation in the following cycle.

10.1128/mbio.01635-22.1 FIG S1 Densitometry analysis of three replicates of Western blot data monitoring egress kinetics of DMSO- and RAP-treated CDC50B. Signals were normalized to the average of the 120-min DMSO-treated time point of maximal signal. A roughly 50% reduction of signal was seen in each time point in RAP-treated parasites. Download FIG S1, PDF file, 0.4 MB. Copyright © 2022 Patel et al. 2022 Patel et al. https://creativecommons.org/licenses/by/4.0/ This content is distributed under the terms of the Creative Commons Attribution 4.0 International license.

In Toxoplasma gondii, the orthologue of CDC50B is required for correct subcellular trafficking of TgGC (22). To investigate whether this is also true in P. falciparum, we used a CRISPR-Cas9-based approach to fuse GCα to a C-terminal mCherry tag in the CDC50B-HA:loxP line, creating a parasite line called CDC50B-HA:loxP GCα-mCherry (Fig. S2). We failed to detect the tagged protein directly by fluorescence microscopy, possibly due to the previously reported very low abundance of GCα (18). However, Western blotting revealed an ~250-kDa signal in extracts of the CDC50B-HA:loxP GCα-mCherry schizonts, likely representing a proteolytic fragment of the tagged protein, since GCα is prone to proteolytic degradation in both P. falciparum and T. gondii (18, 22) (Fig. 4C). Interestingly, Western blotting of extracts of RAP-treated CDC50B-HA:loxP GCα-mCherry schizonts indicated that there was no detectable reduction in the levels of GCα in the absence of CDC50B (Fig. 4C and Fig. S3A). Exploiting the tagged GCα-mCherry line, we sought to confirm whether CDC50B was coprecipitated when GCα-mCherry was immunoprecipitated using red fluorescent protein (RFP)-trap beads which bind mCherry. Coprecipitation of CDC50B was observed, confirming CDC50B binding by GCα-mCherry (Fig. S4).

10.1128/mbio.01635-22.2 FIG S2 Schematic representation outlining the generation of CDC50B-HA3:loxP GCα mCherry by CRISPR-mediated homologous recombination. CDC50B-HA3:loxP was transfected with donor DNA and three Cas9 gRNA plasmids that target the region downstream of the second guanylyl cyclase catalytic domain (C2). Yellow lollipops represent translational stop codons, light blue boxes indicate regions of recodonized sequence, red boxes represent mCherry sequence, orange boxes represent a T2A skip peptide sequence, and green boxes represent the blasticidin marker (BSD) used for selection of integrants. Screening PCRs are represented by double-headed arrows. Right panel, PCR screening of gDNA to validate integration. Left, screening of control 3D7DiCre parental gDNA; right, gDNA taken from transfected parasites that had been selected with BSD. Lane C is a control genomic locus (p230p) to normalize for DNA quality; lanes 1, 2, and 3 represent PCRs 1, 2, and 3. Expected size products for each PCR are as follows: control (primers 35 and 36 [Table 1]), 1,011 bp; PCR 1 (primers 30 and 34 [Table 1]), 1,894 bp; PCR 2 (primers 32 and 33 [Table 1]), 843 bp; PCR 3 (primers 31 and 34 [Table 1]), 1,113 bp. Download FIG S2, PDF file, 0.4 MB. Copyright © 2022 Patel et al. 2022 Patel et al. https://creativecommons.org/licenses/by/4.0/ This content is distributed under the terms of the Creative Commons Attribution 4.0 International license.

10.1128/mbio.01635-22.3 FIG S3 (A) Densitometry analysis of the expression of GCα-mCherry in the presence (DMSO) and absence (RAP) of CDC50B. The signal for duplicate experiments from Fig. 4C was analyzed. The band intensity for GCα-mCherry was normalized to that of the GAPDH loading control to give a relative expression value. The means of two independent experiments are plotted. Error bars, SD; n.s, not significant by Student's *t* test. (B) Densitometry analysis of the recovery of egress in zaprinast- and PET-cGMP-treated CDC50B parasites. Relative signal intensity is plotted for three independent experiments of Fig. 4F. Signals were normalized to the average of the 60-min DMSO-treated parasites. Download FIG S3, PDF file, 0.4 MB. Copyright © 2022 Patel et al. 2022 Patel et al. https://creativecommons.org/licenses/by/4.0/ This content is distributed under the terms of the Creative Commons Attribution 4.0 International license.

10.1128/mbio.01635-22.4 FIG S4 Immunoprecipitation of GCα-mCherry. Samples were loaded in duplicate and probed for mCherry (left panel) and HA epitope (right panel). An asterisk denotes the ~40-kDa degradation product of GCα-mCherry observed after enrichment and boiling of the RFP-trap beads, suggesting that GCα was prone to proteolysis under the conditions used to promote binding or degrades when heated. Download FIG S4, PDF file, 0.2 MB. Copyright © 2022 Patel et al. 2022 Patel et al. https://creativecommons.org/licenses/by/4.0/ This content is distributed under the terms of the Creative Commons Attribution 4.0 International license.

To examine the role of CDC50B in trafficking of GCα, we used an anti-mCherry antibody to localize GCα-mCherry by IFA in RAP-treated CDC50B-HA:loxP GCα-mCherry parasites. This revealed no obvious mislocalization of GCα in the absence of CDC50B, with a similar, diffuse signal detectable in both RAP- and DMSO-treated schizonts. In addition, in contrast to previous findings with T. gondii (22), no mislocalization of GCα in the endoplasmic reticulum (ER) or secretory pathway in the absence of CDC50B was detected, as judged by colocalization with the ER marker plasmepsin V (PMV) (Fig. 4D). Taken together, these results indicate that CDC50B binding is not important for the correct trafficking or stable expression of GCα. To seek more insight into the egress defect, we investigated whether ablation of CDC50B resulted in changes in cyclic nucleotide levels. To do this, we assayed extracts of DMSO- and RAP-treated CDC50B-HA:loxP schizonts by ELISA to quantitate cGMP and cAMP levels. This showed that CDC50B null parasites contained 53.67% (±12.16%) less cGMP than DMSO-treated controls, while no significant difference in cAMP levels was observed (Fig. 4E). These reduced cGMP levels suggested that binding of CDC50B to GCα might be required for maximal GCα cyclase activity. To test this, we investigated whether the defect in egress of CDC50B null schizonts could be reversed by the addition of compounds that stimulate or mimic elevated cGMP levels. Egress was monitored in the presence and absence of the PDE inhibitor zaprinast or PET-cGMP, a membrane-permeable cGMP analogue known to activate parasite PKG (18, 36). Treatment with either compound restored egress of CDC50B null schizonts to levels similar to those observed in control CDC50B-HA:loxP schizonts, confirming the requirement of CDC50B for optimal cGMP synthesis by GCα (Fig. 4F and Fig. S3B).

In view of the above results, as well as the previous observation that a lipid cofactor may stimulate egress in *Toxoplasma* and P. falciparum (22, 37), we examined whether the potential phospholipid flippase activity of the P4-ATPase domain of GCα might be modulated by CDC50B binding. To do this, we investigated whether uptake of fluorescently labeled phosphatidylserine (PS), phosphatidylethanolamine (PE), and phosphatidylcholine (PC) were affected following disruption of CDC50B. DMSO- or RAP-treated CDC50B-HA:loxP late schizonts were incubated with fluorescent lipids and then analyzed by flow cytometry to determine their ability to accumulate lipids. We found no significant difference in the bulk uptake of measured lipids in schizonts in the presence or absence of CDC50B (Fig. S6).

10.1128/mbio.01635-22.5 FIG S5 Gating strategy used to produce histograms of populations of fluorescent lipid-labeled parasites. Cells were first gated for all nonoutlier events (panel 1), followed by gating for single cells (panel 2). Single cells were gated for Hoechst signal, and high-Hoechst-signal cells gate in R1 (panel 3). R1 gated cells were further analyzed for lipid fluorescence, and high fluorescence signal was gated in R2 (panel 4). This gate was set such that it is above signal seen from lipid staining of uninfected erythrocytes. The cells selected by gating in R2 were then plotted as a histogram (panel 5). Download FIG S5, PDF file, 0.2 MB. Copyright © 2022 Patel et al. 2022 Patel et al. https://creativecommons.org/licenses/by/4.0/ This content is distributed under the terms of the Creative Commons Attribution 4.0 International license.

10.1128/mbio.01635-22.6 FIG S6 Flow cytometry analysis of fluorescent lipid uptake in CDC50CB WT (DMSO) and null (RAP) late schizonts. Histograms are overlaid for a count of 10,000 cells for each treatment. Cells were gated for DNA content and further for green fluorescence. A small shift in histogram curves was seen for NDB-PS and NBD-PC for RAP-treated parasites in one of three independent experiments (result shown); however, this result was not reproducible, and no shift was observed in the two subsequent experiments. Control samples, with no lipid added, were run to validate the gating protocol for lipid signal. Right panel, examples of the stained cells visualized by fluorescent microscopy. Download FIG S6, PDF file, 0.2 MB. Copyright © 2022 Patel et al. 2022 Patel et al. https://creativecommons.org/licenses/by/4.0/ This content is distributed under the terms of the Creative Commons Attribution 4.0 International license.

### CDC50C is not required for lipid uptake or protein export but plays an essential role in hemoglobin uptake from the host erythrocyte.

**FIG 5:**
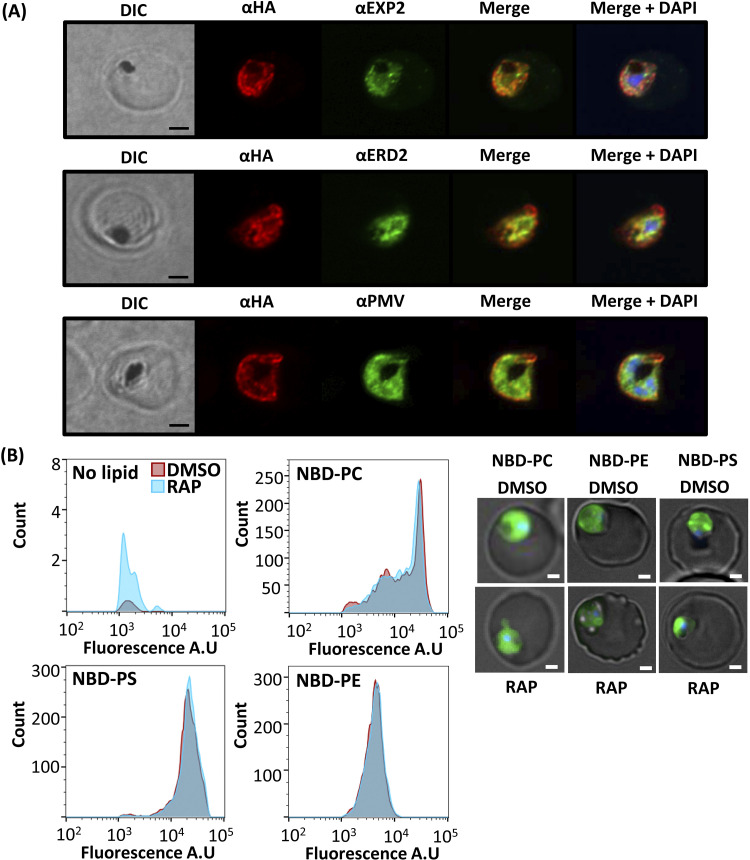
(A) Airyscan confocal analysis of IFA of CDC50C-HA:loxP trophozoites costained with EXP2 (PF3D7_1471100), an exported PV protein; ERD2, a Golgi marker (PF3D7_1353600); and PMV, an ER marker (PF3D7_1323500). Scale bar, 2 μm. (B) Flow cytometry analysis of fluorescent lipid uptake in live WT (DMSO) and CDC50C null (RAP) trophozoites labeled at 36 h postinvasion. Histograms are overlaid, each representing 10,000 cells for each treatment. Cells were gated for DNA content and for green fluorescence. No detectable shift in histogram curves was seen for each lipid in RAP-treated samples. Data are representative of one of three independent experiments, each of which showed the same outcome. Control samples, with no lipid added, were analyzed to validate the gating protocol for lipid signal. Right panel, examples of the stained cells visualized by fluorescence microscopy. Scale bar, 2 μm.

As described above, RAP treatment of synchronous, newly invaded CDC50C-HA:loxP rings produced a CDC50C null parasite population that displayed a trophozoite arrest phenotype (Fig. 2B), suggesting an essential role for CDC50C in the trophozoite-to-schizont transition. Given this evidence that CDC50C plays a very different role from that of CDC50B, we decided to interrogate more precisely the functional role of CDC50C. Previous transcriptomic analysis has shown that CDC50C is transcribed throughout the asexual blood stage cycle, with relatively low levels of transcription in rings increasing to a peak in mature schizont stages (34). Consistent with this transcriptional profile, immunostaining of CDC50C-HA:loxP parasites detected expression in ring, trophozoite, and schizont stages (Fig. S7). Costaining of CDC50C in trophozoites with antibodies to ERD2 (a Golgi marker), PMV (an ER marker), or EXP2 (a PVM marker) showed that CDC50C displayed a diffuse cytosolic staining (Fig. 5A).

10.1128/mbio.01635-22.7 FIG S7 IFA images of CDC50C-HA:loxP parasites throughout the asexual cycle. Scale bar, 2 μm. Download FIG S7, PDF file, 0.5 MB. Copyright © 2022 Patel et al. 2022 Patel et al. https://creativecommons.org/licenses/by/4.0/ This content is distributed under the terms of the Creative Commons Attribution 4.0 International license.

Phospholipid flippases have been shown to contribute directly to cellular lipid uptake (38–40). Initially, we speculated that the growth arrest of CDC50C null trophozoites may be due to a dysregulation of lipid uptake as a result of loss of function of the putative aminophospholipid flippase ATP2 partner. To test this notion, we labeled live RAP- and DMSO-treated CDC50C-HA:loxP trophozoites with the fluorescent aminophospholipid analogues nitrobenzoxadiazol (NBD)-PC, NBD-PE, and NBD-PS. Microscopy showed that lipid labeling was observed predominantly in parasites and not the erythrocyte membrane (Fig. 5B, right). No discernible difference in lipid uptake between CDC50C null and WT trophozoites was observed by flow cytometry (Fig. 5B), suggesting that CDC50C plays no essential role in the uptake of these phospholipids.

**FIG 6:**
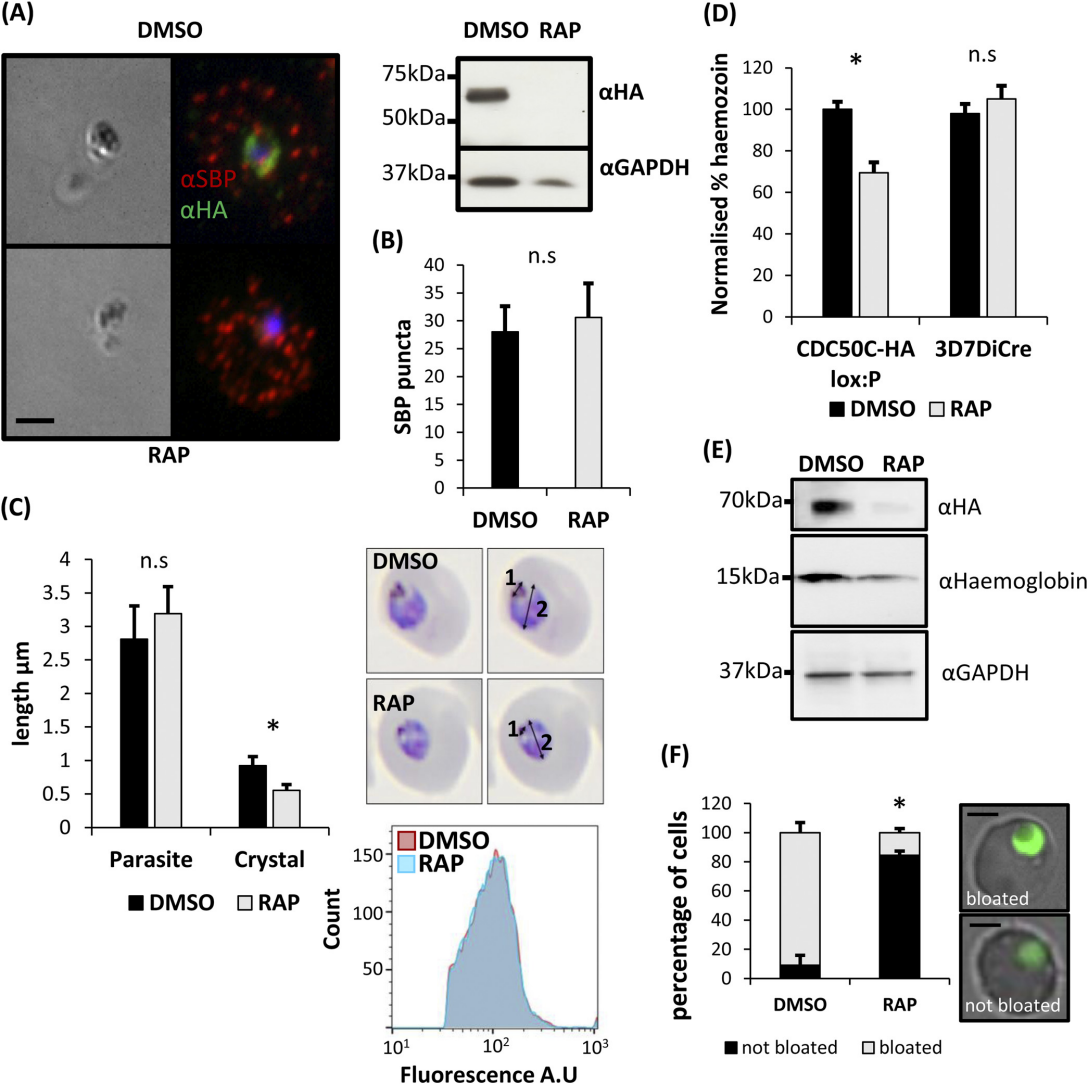
(A) IFA imaging of DMSO- and RAP-treated CDC50C-HA:loxP trophozoites fixed at 36 h postinvasion indicates no defect in export of skeleton binding protein (SBP). Right, Western blot showing absence of CDC50C-HA in RAP-treated trophozoites. Scale bar, 2 μm. (B) Quantification of SBP puncta in DMSO- and RAP-treated CDC50C-HA:loxP trophozoites. Sixty-six parasites were counted from two independent experiments for SBP puncta in ImageJ. Mean values are plotted. Error bars, SD; n.s, not significant, Student's *t* test. (C) CDC50 null parasites produce smaller hemozoin crystals. Thin blood films were made from tightly synchronized DMSO- and RAP-treated CDC50C-HA:loxP parasites at 36 h postinvasion. Inset, length of the hemozoin crystal (measurement 1) and parasite (measurement 2) were performed in ImageJ on imaged Giemsa-stained smears of DMSO- and RAP-treated CDC50C-HA:loxP trophozoites. In total, 60 control or RAP-treated parasites were measured from three independent experiments. Mean values are plotted. Error bars, SD; *, *P* < 0.05, Student's *t* test. (D) Spectrophometric quantification of the effects of CDC50C ablation on parasite hemozoin content. Highly synchronized ring stage CDC50C-HA:loxP cultures were treated with DMSO (control) or RAP. Cultures were harvested at 36 h postinvasion, and hemozoin was purified using established methods (45) and then quantified by absorbance at 410 nm. Means are plotted for three independent experiments. Error bars, SD; n.s, not significant; *, *P* < 0.05, Student's *t* test. (E) Western blot analysis of the effects of CDC50C ablation on hemoglobin content. Highly synchronized ring stage CDC50C-HA:loxP cultures were treated with DMSO (control) or RAP. Cultures were then harvested at 36 h postinvasion, and parasites were released using saponin. Parasite extracts were probed for the presence of CDC50C by HA staining. Hemoglobin content was probed alongside GAPDH as a loading control. Data are representative of three independent experiments. (F) Effects of CDC50C ablation on E64-mediated food vacuole bloating. Tightly synchronized DMSO- or RAP-treated CDC50C-HA:loxP parasites were treated with 33 μM E64 at 24 h postinvasion and left to develop for a further 8 h, after which they were stained with 4.5 μg/mL dihydroethidium to detect the food vacuole and imaged. A minimum of 20 cells were counted per condition and scored for bloated or nonbloated food vacuoles. An inset shows representative images of bloated and nonbloated parasites; scale bar, 2 μm. Mean data are plotted for three independent experiments. Error bars, SD; *, *P* < 0.05, Student's *t* test for the comparison of nonbloated parasites between DMSO and RAP treatments.

In model organisms, flippases also contribute to the production and maintenance of membrane asymmetry required for generation of trafficking vesicles, with specific flippases influencing exocytosis or endocytosis pathways (39, 41–43). Given that lipid uptake was unaffected in the absence of CDC50C, we considered it plausible that the ATP2-CDC50C complex may contribute to lipid homeostasis and trafficking in an analogous manner. P. falciparum trophozoites remodel their intracellular environment to create new permeation pathways that enable export of a wide variety of proteins into the host RBC via exocytosis, a process which is essential for trophozoite development (44). To examine whether trophozoite death in CDC50C null parasites could be attributed to changes in protein exocytosis, control or RAP-treated CDC50C-HA:loxP ring stage parasites were allowed to develop into trophozoites and were then analyzed by IFA to determine the localization of skeleton binding protein (SBP), a prominent exported protein in trophozoites. Puncta of SBP, characteristic of export, were evident within the RBC cytosol in both DMSO- and RAP-treated CDC50C-HA:loxP trophozoites (Fig. 6A), and quantification of these puncta indicated that there was no significant change in the levels of export of SBP in CDC50C null trophozoites (Fig. 6B). This suggested that loss of CDC50C has no impact on bulk protein export during trophozoite development.

As these results suggested that protein export and exocytosis were unaffected in CDC50C null trophozoites, we examined an essential endocytotic process. As intracellular asexual blood stage malaria parasites develop, they endocytose and digest host erythrocyte hemoglobin. A major by-product of this catabolic process is the sequestration of heme in the form of a characteristic crystalline product called hemozoin, which accumulates in the parasite digestive vacuole as large, refractile complexes that are easily visible by light microscopy. Microscopic examination of Giemsa-stained thin blood films indicated that while CDC50C null trophozoites displayed an apparently normal morphology, the hemozoin crystals appeared smaller than those of DMSO-treated controls (Fig. 6C, right). To examine this in greater detail, we compared the ratio of the hemozoin crystal length to that of the parasite length in RAP- and DMSO-treated CDC50C null trophozoites. This confirmed a significantly decreased size of hemozoin in CDC50C null trophozoites, whereas parasite size was unaffected (Fig. 6C), suggesting a CDC50C-dependent defect in hemozoin formation. To further examine this, we purified and quantified hemozoin from parallel populations of RAP- and DMSO-treated CDC50C-HA:loxP trophozoites (45). As shown in Fig. 6D, this revealed ~30% less hemozoin in trophozoites lacking CDC50C. To investigate whether uptake of hemoglobin was affected in CDC50C null trophozoites, RAP- and DMSO-treated CDC50C-HA:loxP trophozoites were harvested at 36 h postinvasion and released from their host cells using saponin, and levels of intraparasite hemoglobin were quantified by Western blotting (Fig. 6E). The results indicated that the levels of hemoglobin within CDC50C null trophozoites were significantly reduced compared with those of control counterparts (Fig. S8). We further interrogated the role of CDC50C in the uptake of hemoglobin by using a food vacuole bloating assay by treatment of trophozoites with the cysteine protease inhibitor E64 (46). We found that while WT parasites displayed the characteristic bloating phenotype observed upon inhibition of hemoglobin digestion, RAP-treated parasites were largely unaffected (Fig. 6F), suggesting that these parasites are defective in hemoglobin transport to the food vacuole. Collectively, these data support a role for CDC50C in uptake and digestion of host erythrocyte hemoglobin that is essential for parasite development.

10.1128/mbio.01635-22.8 FIG S8 Densitometry analysis of three sets of independent Western blot data monitoring hemoglobin uptake in CDC50C DMSO- and RAP-treated trophozoites. Signals were normalized to the average of the DMSO-treated samples. A roughly 70% reduction of signal was seen for HA and hemoglobin in RAP-treated parasites. Download FIG S8, PDF file, 0.4 MB. Copyright © 2022 Patel et al. 2022 Patel et al. https://creativecommons.org/licenses/by/4.0/ This content is distributed under the terms of the Creative Commons Attribution 4.0 International license.

## DISCUSSION

In this study, we have shown that CDC50B and CDCD50C proteins are expressed during asexual blood stage development and that they each bind to different putative P4-ATPase flippases—GCα and ATP2, respectively—which function at different developmental stages of the asexual blood stage cycle. CDC50B is dispensable for intraerythrocytic development, as is its orthologue in P. yoelii (20). Consistent with this, CDC50B null parasites display normal intracellular maturation and no significant replication defect over multiple erythrocytic cycles. However, we find that cGMP levels are reduced in CDC50B null parasites as well as rates of egress following schizont maturation. This effect is likely due to a delay in PKG activation, as this defect can be rescued by treatment of CDC50B null parasites with either the PDE inhibitor zaprinast or PET-cGMP, a membrane-permeable cGMP analogue known to be capable of activating apicomplexan PKGs (18). Collectively, our results strongly suggest that CDC50B acts to enhance cGMP synthesis by GCα. Recent studies in T. gondii have shown that the single GC (TgGC) also binds to a CDC50 designated CDC50.1 (22). We find that CDC50B is phylogenetically most closely related to CDC50.1 and CDC50.2 (see Fig. S9A in the supplemental material). In contrast to the present study, knockdown of CDC50.1 resulted in mislocalization of TgGC and a block in egress of T. gondii tachyzoites (22). The egress block could be rescued by adding a PDE inhibitor, implying that in the absence of CDC50.1, TgGC remains functional but produces cGMP with reduced efficiency (22). We observed no detectable mislocalization of P. falciparum GCα in the absence of CDC50B, with normal growth of parasites despite the reduced egress rates, indicating differences between the genera. We speculate that this may be due to differences in the threshold levels of cGMP required to activate PKG to trigger egress in each species.

10.1128/mbio.01635-22.9 FIG S9 (A) Clade tree comparing CDC50s from P. falciparum and T. gondii. (B) Alignment of P. falciparum CDC50A (PlasmoDB identifier PF3D7_0719500), CDC50B (PlasmoDB identifier PF3D7_1133300), and CDC50C (PlasmoDB identifier PF3D7_1029400) with S. cerevisiae Cdc50p (UniProt accession no. P25656) and LEM3 (UniProt accession no. P42838) and human cdc50a (UniProt accession no. Q9NV96). The alignment shows the region containing the conserved Asn180 residue in human cdc50a highlighted in blue, at which glycosylation has been shown to interact structurally with its partner P4-ATPase (28). This residue is replaced with a Thr in CDC50B. Red residues are identical in S. cerevisiae and Homo sapiens sequences and *Plasmodium* CDC50 sequences. Yellow highlighted numbers indicate insertions of amino acids of specified length. Two conserved cysteine residues are highlighted in grey. (C) Alignment of P. falciparum ATP2 (PlasmoDB identifier PF3D7_1219600) with P. falciparum ATP7 (PlasmoDB identifier PF3D7_0319000), human ATP8A1 (UniProt accession no. Q9Y2Q0-2), human ATP8B1 (UniProt accession no. O43520), yeast Drs2p (UniProt accession no. P39524), and yeast Dnf1 (UniProt accession no. P32660) P4-ATPases to highlight functionally important residues, and also with the two Toxoplasma gondii ATP2 paralogues, ATP2A (ToxoDB identifier TGME49_216380) and ATP2B (ToxoDB identifier TGME49_247690). Also included for comparison are the P4-ATPase-like domains of the P. falciparum GCα (PlasmoDB identifier PF3D7_1138400) and the T. gondii GC (ToxoDB identifier TGME49_254370). The names of yeast/mammalian P4-ATPases that flip PS are green, and those that flip PC are amber. Red residues are identical or similar (e.g., R,K,H; D,E; L,I,V,A,M; F,Y,W; N,Q,S,T; G,P) in two or more of the apicomplexan ATP2 sequences and three or more of the mammalian and yeast sequences. Amino acids associated with selectivity for PS or PC flipping in human and yeast PS-ATPases are shown in green and amber, respectively. The positions of amino acids shown to be important for phospholipid binding/translocation in a crystal structure of human ATP8A1 (PMID 31416931) are indicated with red circles above the sequence. The important ATP-binding amino acids of the ATP8A sequence (which are all conserved in PfATP2) are F534 in the N-domain that contacts the adenine ring and the phosphate group that interacts with D409 and T411 in the DKTG motif containing the phosphorylation site (D409) and with N789 and D790 toward the end of the P domain, along with a Mg^2+^ ion. The strong association of the A-domain with the phosphorylation site in the human ATP8A crystal structure is mediated by D189 and G190 in the DGET motif, which is again conserved in PfATP2. In accordance with the scheme depicted in the structural study of human ATP8A1, the actuator domain (A-domain) sequence is shown in an orange box, the phosphorylation domain (P-domain) sequence is shown in a blue box, and the nucleotide-binding domain (N-domain) sequence is shown in a pink box. Important functional motifs are indicated above the sequence and boxed. Transmembrane domains are indicated above the sequence by green bars, and the corresponding sequences are highlighted in grey. Amino acids that are identical in the four human and yeast P4-ATPases as well as P2-ATPases (SERCA Ca^2+^ ATPase [UniProt accession no. P04191] and Na^+^/K^+^ ATPase [UniProt accession no. Q4H132], not included in the alignment) are indicated in bold red. Amino acid numbers are shown to the right of each sequence. Download FIG S9, PDF file, 0.7 MB. Copyright © 2022 Patel et al. 2022 Patel et al. https://creativecommons.org/licenses/by/4.0/ This content is distributed under the terms of the Creative Commons Attribution 4.0 International license.

Importantly, our work adds to the evidence supporting a role for CDC50s and the P4-ATPase domain of apicomplexan GCs acting functionally to stimulate maximal cGMP production required for egress. By analogy with other CDC50-flippase interactions, we speculate that this occurs through CDC50B binding to the P4-ATPase domain of GCα. Modulation of the activity of the C-terminal cyclase domain by the P4-ATPase may integrate a lipid-mediated trigger for egress, potentially by phosphatidic acid, as shown in T. gondii (22), or phosphatidylcholine (PC), as recently indicated in P. falciparum (37). However, we did not observe changes in the bulk uptake of fluorescent PC by schizonts lacking CDC50B. Recent structural examination of a human P4-ATPase:CDC50 complex has shown that CDC50 forms an intimate interaction with the TMDs of the P4-ATPase partner, with the loop domain between the two TMDs of the CDC50 forming an antiparallel beta-sheet structure that contacts the luminal side of the transmembrane loops of the P4-ATPase. Human CDC50 is glycosylated at several conserved asparagine residues, and the structure showed that interactions between CDC50 glycan moieties and P4-ATPase stabilize the functional complex (28). Intriguingly, an alignment of human CDC50a and the three P. falciparum CDC50s indicates that Asn180, at which glycosylation has been shown to interact structurally with its partner P4-ATPase (28), is absent from CDC50B but conserved in both CDC50A and CDC50C (Fig. S9B). N-glycosylation has been observed in P. falciparum (47), but this finding raises the possibility that CDC50B may be nonglycosylated. This observation may explain the finding in P. yoelii that GCβ is degraded in the absence of its partner CDC50, suggesting that GCβ is highly reliant on interactions with its CDC50 partner (CDC50A) for protein stabilization (20). In contrast, in our study, we observe that loss of CDC50B does not impact expression or stability of GCα, since GCα-mediated egress still occurs.

The revelation that CDC50C is essential for intraerythrocytic maturation of asexual blood stage P. falciparum trophozoites and that CDC50C binds to ATP2 suggests that CDC50C plays a role critical for ATP2 function. In contrast to our findings using native parasite-derived protein preparations, a recent *in vitro* study using recombinant protein indicated that ATP2 can bind CDC50B; however, CDC50C binding was not tested in that work, as the authors could not express it (48). Our study indicates that the essential function of CDC50C cannot be complemented by CDC50B.

Global transposon mutagenesis data suggest that the gene encoding ATP2 is essential in P. falciparum blood stages (49) and its orthologue is refractory to targeted deletion in Plasmodium berghei (50). While its cellular function is unknown, ATP2 has been implicated in resistance to two Medicines for Malaria Venture (MMV) “Malaria Box” compounds, mediated through a novel pathway involving gene copy number amplification. Functional characterization of the mechanism by which drug resistance is achieved remains lacking (51). Interestingly, Cowell et al. observed nonsynonymous mutations in genes encoding putative parasite Sec24 and Yip1 proteins (classically involved in vesicular trafficking) in drug-resistant parasite lines containing *ATP2* copy number variations (51). Here, we found that the ATP2-CDC50C complex influences endocytosis of hemoglobin during blood stage development possibly by influencing the phospholipid makeup of the cytostome, a structure that is crucial for hemoglobin uptake (52, 53), and it remains possible that other endocytic pathways may also be affected by loss of ATP2 function, although these were not investigated. In yeast, different P4-ATPases contribute to distinct vesicular trafficking pathways (39, 43). It is plausible that this could be similar in P. falciparum. We speculate that copy number modulation of *ATP2* acquired during selection for drug resistance may modulate the endocytic pathway of the parasite so as to affect drug uptake, although further work is required to investigate this.

A recent study in P. yoelii has shown that the orthologue of CDC50C binds to a different P4-ATPase (ATP7) in ookinetes during parasite development within the mosquito (54). This indicates that CDC50C chaperones the activity of distinct P4-ATPases in different developmental stages of the parasite life cycle in both mammalian and insect hosts. Consistent with this, the transcriptomic profiles of *ATP2* and *ATP7* show that they are confined to asexual and insect stages, respectively. The same study demonstrated that the ATP7-CDC50C complex is required for PC uptake in ookinetes, and the authors suggested that this process may be required to allow mosquito midgut cell traversal, as CDC50C null or ATP7 null ookinetes could not achieve this. Intriguingly, alignment of ATP7 and ATP2 primary sequences alongside those of model P4-ATPases revealed that the “QQ motif” involved in defining substrate specificity is replaced by QL and QV, respectively (Fig. S9C). Given the similarity between these amino acid motifs, it is plausible that ATP2 also transports PC. Our finding that NBD-PC uptake is unaffected in CDC50C null trophozoites suggests that either ATP2 transports another phospholipid or that lipid uptake in trophozoites occurs via (multiple) redundant pathways. During the preparation of this article, a recent study found that the T. gondii orthologue of CDC50C, CDC50.4, binds ATP2B, an essential P4-ATPase that transports PS (55). This CDC50.4-ATP2B complex is required for efficient microneme secretion in tachyzoites with no defect observed during parasite intracellular development (55). It is plausible that the P. falciparum CDC50C-ATP2 complex may perform a similar role in egressed merozoites, but this could not be addressed in our study due to the block in intraerythrocytic development in CDC50C null parasites. Our work provides substantial new insights into the multifaceted, essential roles played by CDC50C proteins in malaria parasites and highlights potential species-specific differences between the roles of CDC50s in apicomplexan parasites.

## MATERIALS AND METHODS

### P. falciparum culture and synchronization.

P. falciparum erythrocytic stages were cultured in human erythrocytes (National Blood Transfusion Service, United Kingdom) and RPMI 1640 medium (Life Technologies) supplemented with 0.5% Albumax type II (Gibco), 50 μM hypoxanthine, and 2 mM l-glutamine. Synchronous parasite cultures were obtained as described previously (56). Briefly, late segmented schizonts were enriched by centrifugation on a 60% Percoll (GE Healthcare) cushion, followed by the addition of fresh erythrocytes to allow invasion for 1 to 2 h under continuously shaking conditions. The remaining schizonts were then removed by sorbitol treatment to yield highly synchronous ring stage cultures. In all cases, induction of DiCre activity when required was by treatment for 2 to 4 h with 100 nM RAP (Sigma) as described previously (32, 57). Control parasites were treated with vehicle only (1% [vol/vol] DMSO).

### Genetic modification of P. falciparum parasites.

The CDC50A-HA:loxP, CDC50B-HA:loxP, and CDC50C-HA:loxP lines were generated from the DiCre-expressing 3D7 (33) P. falciparum clone using selection-linked integration (SLI) of a plasmid containing a SERA2loxPint (57) followed by a triple-HA tag and an in-frame Thosea asigna virus 2A (T2A) ribosomal skip peptide and NeoR cassette with a downstream loxP and PbDT 3′-untranslated region (UTR) sequences as described previously. Recodonized versions of the C-terminal portion of each gene containing the last transmembrane helix were synthesized commercially (IDT) and inserted downstream of the SERA2loxPint and upstream of the 3×HA tag. Sequences were as follows: CDC50A, GATTTCTGGCTCATGAACGAAAAGTACAAGAACGCATTAAACATGAACAATGAGAACGGTTACGGTGACGAAAACAGTCACTTCATAGTTTGGATGAAGACTGCAGCTTTGAGTGAATTTAGAAAGAAGTACGCAAAGATTAACGTAGAGGTAAACTTGCCTATTTACGTTAACATAAACAACAACTTCCCAGTCACCAAGTTCAACGGAAAGAAGTTCTTCGTAATCGCAGAGGGTAGTATTTTCATTAACGAGAAGATTCAGTCTCTCGGTATTCTCTATTTGGTTATAGGTATAATTAGTCTAGGTATAGTTGCATGCCTTATTTACAACCAGATGAAGAATCCGAGGATAATTGGATATCACGCTTATATTTACATCTTCTTCTTCTTGG; CDC50B, GATCACATTTACTTTTGGATGGAGCCTGATATTCAGTACGAGCGTTTGCAGGAGAACAAGGAGACTAACGAGAAATTGCTAGTTTTGCCTCAGACTTTGAAGTACAACCAGGCTGGTAAGGCAATTGAGAATTCTCACTTCATAAACTGGATGATTCCTAGTGCTCTAAACTACATAAAGCGATTGTACGGAAAGTTGTACATTCCATTGAAGTTCCCCTTCTACATCTACATTGAGAACAACTTCAAGATAAACGACACTAAGATAATCGTAATATCTACATCTCAGTACTACATGAGGACCTTCTTGATCGGCTTTATATTCATCATCATAAGTATCATTGCATTGATCTTGTGCATCTTCTACCTCATCAGGATGAACAAGTACGAGAACAAG; CDC50C, GATGAGTGGAACGCTAAGAAAAGTTTCCAGCTTGTGAGTCTTCGTTCTATTGGTAACTCAAGTTTCAAGTTAGCCTACGCATTCTTTCTTTTAAGTTTGTTGTATTTCATCATGATTATATTCATATTGGTTTTGGTGAAGTGCAAGTACTATAAATTGGGTAAGACTCTTACATACTGTAAGTTATCTATGAACAAGAACATTGAGAAGATGAACTCAAGGAAGAAGACTAACATTCAGAACATTAACAAGAAAATAAACAGTATGCAGCTTGAGATAATGCATAAAGCCTCATCAGATCCTAACAATCTTGCTGGTGCTGACCACAGTCAGAAGTTGTGTTTCTGTCCATTGCATG. An 800-bp 5′-end homology region comprising the native gene sequence upstream of the recodonized region was cloned upstream of the SERA2loxPint. Following transfection of purified schizonts using an Amaxa 4D-Nucleofector system (Lonza) and P3 reagent, modified parasites were selected as described previously (58).

Oligonucleotide primers used in diagnostic PCR to detect integration and excision of transgenes are provided below in Table 1.

**TABLE 1:** Oligonucleotide primers used in this study

Primer	Name	Sequence
1	CDC50A 5HR F	GCGGCCGCAGATCTCTCGAGCGATATTGGACACCAAATTGTTTA
2	CDC50A 5HR R	CGAAGTTATTGTATATTATTTTTTTTATTTACCTACATGTGATTTATGTAATTCCATTTC
3	CDC50A reco F	GTATATATATATATATTTATATATTTTATATTCTTTTAGATTTCTGGCTCATGAACGAAAAGTACAAGAACGCATTAAACATG
4	CDC50A reco R	CAGATCCGCCTGAACCGGATCCCAAGAAGAAGAAGATGTAAATATAAG
5	CDC50B 5HR F	GCGGCCGCAGATCTCTCGAGTGAGTAATCTTAAAAATGACATGTTTATATC
6	CDC50B 5HR R	CGAAGTTATTGTATATTATTTTTTTTATTTACCTTTATATAATTGTACATTTTGAGGTG
7	CDC50B reco F	GTATATATATATATATTTATATATTTTATATTCTTTTAGATCACATTTACTTTTGGATGGAGC
8	CDC50B reco R	CAGATCCGCCTGAACCGGATCCCTTGTTCTCGTACTTGTTCATC
9	CDC50C reco F	GTATATATATATATATTTATATATTTTATATTCTTTTAGATGAGTGGAACGCTAAGAAAAGTTTC
10	CDC50C reco R	CCGCCTGAACCGGATCCATGCAATGGACAGAAACACAACTTC
11	CDC50C 5HR F	GATCTCTCGAGCCAGAGTACGAATTCATGAATGCTTTTAAACAACAAG
12	CDC50C 5HR R	CGAAGTTATTGTATATTATTTTTTTTATTTACCTGCTGGCCATACGTTTTGAAG
13	5HR seq F	CAGCTATGACCATGATTACGCC
14	Reco seq F	CATTATACGAAGTTATTATATATGTATATATATATATATTTATATATTTTATATTC
16	PKAc screen F	GAAGGACAGTGATTCTAGTGAACAG
17	PKAc WT screen R	CAATTTCTTCATCAAATGTTTGCAATTGTTATC
18	HA R	GCATAGTCAGGAACATCGTAAGG
19	Exi R	CCGTTCAAATCTTCTTCAGAAATCAAC
20	3′ int F	CAGCTATGACCATGATTACGCC
21	CDC50A 5′ int F	CTTTAGATTATGATGATAATTTTTTGGAAGAAAAG
22	CDC50A WT R	GTGTATATTTAAAAATCAGGATTTTACTATATCCTC
23	CDC50B 5′ int F	CAGTTATGTGTCTTCCCTTTGTATTATTTTG
24	CDC50B WT R	CTTTTGGTTATTAAATGTGTATCGAAATAATAC
25	CDC50C 5′ int F	GTCGCAGTTCATGGGAAGG
26	CDC50C WT R	GGGAATGGTCTGCTCCTGCT
27	T2A BSD amp F	GCGGCATGGACGAGCTGTACAAGAGTGGAGAAGGAAGAGG
28	T2A BSD amp R	GTGTTGATGGTTTTGGGCTAGCTTAGCCCTCCCACACATAACC
29	Screen T2A F	CGAGGACTACACCATCGTGG
30	GCtag WT F	CTAAGAATATTCATTCCTACGATG
31	GCtag 3′ int F	CAATGGCACCTTTGTCTCAAG
32	GCtag 5′ int F	CATGGGCAAATGGTGTAGATG
33	GCtag 5′ int R	CCTCCATGTGCACCTTG
34	GCtag WT R	CGAATGTTCGGAAAAATATTCATGTGC
35	p230p Int screen F	CTATATGGTATCCAAAACCTTTAAATTATATAGC
36	p230p WT screen R	GAGGAATTTTTAAATATGATATACCTTTATCATTAG

CDC50B-HA:loxP GCα-mCherry was generated by transfection of CDC05B-HA:loxP. A linearized donor DNA which inserted mCherry in-frame with the C terminus of GCα, followed by a T2A peptide and BSD selection marker when integrated, and three pDC2-based (33) Cas9 guide RNA (gRNA) plasmids were cotransfected, each with different single guide RNA (sgRNA) targeting the C terminus of GCα. sgRNA sequences were as follows: sgRNA1, CTCTAAATTATTACAAAATA; sgRNA2, AGAAAAAACATTCAAGTATC; sgRNA3, ACGATGAAAAAAAGAAGAAG. Parasites were left to grow for 2 days posttransfection, followed by treatment with 5 μg/mL BSD to select for integrants. After the emergence of BSD-resistant parasites, gDNA was screened for correct integration. Donor sequences were constructed by amplifying a T2A BSD sequence from pDCIn (DiCre induction) (17) by PCR and cloning using a BsrGI site in frame with the C terminus of a donor DNA targeting GCα which had previously been constructed in the lab (18).

### Parasite sample preparation and Western blotting.

Parasite culture supernatant samples for egress and adhesin shedding assays were prepared from tightly synchronized cultures as previously described (17). Percoll-purified mature schizonts were resuspended in complete medium and allowed to further mature for 3 h until they were predominantly mature segmented schizonts. The experiment was then initiated by washing parasites with RPMI 1640 medium three times, followed by final resuspension at a 10% hematocrit in fresh warm RPMI 1640 medium. Culture supernatant aliquots (100 μL) were harvested at specified time points by centrifugation. The schizont pellet from time zero was retained as a pellet control sample.

Parasite extracts were prepared from Percoll-purified schizonts treated with 0.15% (wt/vol) saponin to remove erythrocyte material. To solubilize parasite proteins, phosphate-buffered saline (PBS)-washed saponin-treated parasite pellets were resuspended in 3 volumes of NP-40 extraction buffer (10 mM Tris, 150 mM NaCl, 0.5 mM EDTA, 1% NP-40, pH 7.5, with 1× protease inhibitors [Roche]). Samples were gently vortexed and incubated on ice for 10 min, followed by centrifugation at 12,000 × *g* for 10 min at 4°C. For Western blotting, SDS-solubilized proteins were electrophoresed on 4% to 15% Mini-Protean TGX Stain-Free protein gels (Bio-Rad) under reducing conditions and proteins were transferred to nitrocellulose membranes using a semidry Trans-Blot Turbo transfer system (Bio-Rad). Antibody reactions were carried out in 1% skimmed milk in PBS with 0.1% Tween 20 and washed in PBS with 0.1% Tween 20. Appropriate horseradish peroxidase-conjugated secondary antibodies were used, and antibody-bound washed membranes were incubated with Clarity Western ECL substrate (Bio-Rad) and visualized using a ChemiDoc (Bio-Rad).

Antibodies used for Western blotting presented in this work were as follows: anti-HA monoclonal antibody (MAb) 3F10 (diluted 1:2,000) (Roche), mouse anti-GAPDH MAb (1:20,000), rabbit anti-SERA5 polyclonal antibody (1:2,000), rabbit anti-mCherry (1:2,000) (Abcam), and rabbit anti-hemoglobin polyclonal antibody (1:2,000) (Sigma). Densitometry quantifications were performed using ImageJ.

### Immunofluorescence assays.

Thin blood films were fixed with 4% formaldehyde in PBS and permeabilized with PBS containing 0.1% (vol/vol) Triton X-100. Blocking and antibody binding were performed in PBS–3% BSA (wt/vol) at room temperature. Slides were mounted with ProLong gold antifade mountant containing DAPI (4′,6-diamidino-2-phenylindole) (Thermo Fisher Scientific). Images were acquired with a Nikon Eclipse Ti fluorescence microscope fitted with a Hamamatsu C11440 digital camera and overlaid in ICY bioimage analysis software or ImageJ. Superresolution images were acquired using a Zeiss LSM880 confocal microscope with an Airyscan detector in Airyscan SR mode. Antibodies used for IFA were as follows: anti-HA monoclonal antibody (MAb) 3F10 (diluted 1:200) (Roche), mouse anti-PMV MAb (1:50), rabbit anti-ERD2 polyclonal antibody (1:2,000), rabbit anti-EXP2 polyclonal antibody (1:500) (Abcam), and rabbit anti-mCherry polyclonal antibody (1:200) (Abcam).

### Flow cytometry.

For growth assays, synchronous ring stage parasites were adjusted to a 0.1% parasitemia–1% hematocrit suspension and dispensed in triplicate into six-well plates. Triplicate samples of 100 μL were harvested at days 0, 2, 4, and 6 for each well and fixed with 4% formaldehyde–0.2% glutaraldehyde in PBS. Fixed samples were stained with SYBR green and analyzed by flow cytometry.

For the measurement of egress and ring formation of highly synchronized DMSO- and RAP-treated CDC50B-HA:loxP parasites, a culture of CDC50B-HA:loxP with a 1-h invasion window was seeded in duplicate at 1% hematocrit and 2% parasitemia and treated with DMSO or RAP at 4 h postinvasion for 2 h. Triplicate samples of 100 μL were taken and fixed with 4% formaldehyde–0.2% glutaraldehyde in PBS at hourly intervals from 45 to 53 h postinvasion and at 69 h postinvasion the subsequent day. Fixed samples were stained with SYBR green and analyzed by flow cytometry. Schizont parasitemia was determine by gating high-signal SYBR-positive cells. Ring parasitemia was determined similarly but by gating low-signal SYBR-positive cells.

### Fluorescent lipid labeling.

NBD-PC, NBD-PE, and NBD-PS (Avanti polar lipids) were dried and resuspended in RPMI 1640 medium to 1 mM stock solutions and stored at –20°C. Relevant parasite stages (trophozoites or late schizonts) from highly synchronous cultures were pelleted and washed twice with RPMI 1640 medium. Parasites were then resuspended in RPMI 1640 medium containing Hoechst stain with 1 μM NBD lipid or no lipid (negative control). Suspensions were incubated at 37°C for 30 min and subsequently pelleted by centrifugation. Pellets were then washed three times with prewarmed RPMI 1640 medium containing 5% bovine serum albumin (BSA), followed by resuspension in PBS. Suspensions were then diluted 1:10 and analyzed by flow cytometry on an Attune NxT. Samples were gated for Hoechst DNA positivity, and the resultant population was gated for NBD lipid fluorescence. This gate was set to exclude signal generated from staining uninfected erythrocytes. For trophozoite samples, a low Hoechst signal population was gated, and for schizont samples, a high Hoechst signal population was gated.

### Immunoprecipitation.

Tightly synchronized schizonts (~45 h old) of CDC50B-HA:loxP, CDC50B-HA:loxP GCα-mCherry, CDC50C-HA:loxP, and 3D7DiCre parental parasites were enriched on a 70% Percoll cushion. The schizonts were treated for 3 h with 1 μM C2 (to arrest egress), after which the cultures were treated with 0.15% saponin in PBS containing cOmplete Mini EDTA-free protease and PhosSTOP phosphatase inhibitor cocktails (both from Roche) for 10 min at 4°C to lyse the host erythrocytes. Samples were washed twice in PBS containing protease and phosphatase inhibitors and snap-frozen, and the pellets were stored at −80°C. Parasite pellets (70- to 100-μL packed volume) were resuspended in 3 volumes of NP-40 extraction buffer (10 mM Tris, 150 mM NaCl, 0.5 mM EDTA, 1% NP-40, pH 7.5, with 1× protease inhibitors [Roche]). Samples were gently vortexed and incubated on ice for 10 min, followed by centrifugation at 12,000 × *g* for 10 min at 4°C. Clarified lysates were then added to anti-HA antibody-conjugated magnetic beads (Thermo Scientific) or RFP-trap beads (ChromoTek) which had been equilibrated in NP-40 extraction buffer. Samples were incubated at room temperature for 2 h on a rotating wheel, after which beads were precipitated using a magnetic sample rack. The supernatant was removed, and beads were washed three times with NP-40 extraction buffer, followed by three washes with extraction buffer lacking detergent. Washed beads were then resuspended in trypsinization buffer [50 mM ammonium bicarbonate, 40 mM 2-chloroacetamide, and 10 mM Tris-(2-carboxyethyl) phosphine hydrochloride], and samples were reduced and alkylated by heating to 70°C for 5 min. Two hundred fifty nanograms of trypsin was added to the samples and heated at 37°C overnight with gentle agitation, followed by filtration using a 0.22-μm Costar Spin-X centrifuge tube filter (Sigma). Samples were then run on a LTQ Orbitrap Velos mass spectrometer (Thermo Scientific). The search engines Mascot (http://www.matrixscience.com/) and MaxQuant (https://www.maxquant.org/) were used for mass spectrometry data analysis. The PlasmoDB database was used for protein annotation. Peptides and proteins having a minimum threshold of 95% were used for further proteomic analyses, and peptide traces were analyzed using Scaffold5. Enrichment was determined by comparing results from tagged lines with that of immunoprecipitated material from 3D7DiCre parental parasites.

### Measurement of hemozoin content.

A culture of 5% parasitemia 1-h synchronized ring stage CDC50C parasites were treated at 1 h postinvasion with DMSO or RAP (100 nM) and then left to develop until the early trophozoite stage at 36 h postinvasion. Parasites were then harvested by saponin lysis and processed similarly to a reported method (45) to purify the hemozoin. Pellets were then depolymerized in 0.5 mL of 0.2 M NaOH solution, and the resultant heme content was measured by absorbance at 410 nm in a Spectramax iD5 plate reader.

### Measurement of intracellular cyclic nucleotide levels.

cAMP and cGMP in mature CDC50B schizonts were measured using enzyme-linked immunosorbent assay (ELISA)-based high-sensitivity direct cAMP and cGMP colorimetric assay kits (Enzo Life Sciences). Mature schizonts were Percoll purified from RAP- or DMSO-treated CDC50B-HA:loxP cultures, followed by resuspension and lysis in 0.1 M HCl solution. Samples were pelleted at 10,000 × *g*, and the supernatant was collected and stored at −80°C until required. To perform the ELISA, samples and standards were acetylated to improve the detection sensitivity according to the manufacturer’s instructions. Standards and samples were run in triplicate on the same plate, and absorbance at 410 nm was read with a SpectraMax iD5 plate reader. The standard was fitted to a sigmoidal curve and used to determine cyclic nucleotide concentrations in parasite samples. The remaining supernatant was assayed for protein concentration by a Bradford assay kit (Pierce). cGMP and cAMP reading were normalized by protein content from the Bradford assay.

### Food vacuole bloating assay.

We conducted experiments similarly to those previously reported (46). CDC50C-HA:loxP parasites with a 1-h invasion window were treated with DMSO or RAP at 1 h postinvasion for 2 h. At 24 h, 33 μM E64 was added and the parasites were left to develop for a further 8 h, after which they were stained with 4.5 μg/mL dihydroethidium for 20 min at room temperature to detect the food vacuole. Parasites were then washed twice with PBS and imaged on an EVOS fluorescence microscope. A minimum of 20 cells were counted per condition and scored for bloated or nonbloated food vacuoles.

### Data availability.

Data are publicly available via the PRIDE database under data set identifier PXD033834.
