# Supplementary figures and images for: CDC50 Orthologues in Plasmodium falciparum Have Distinct Roles in Merozoite Egress and Trophozoite Maturation
Source: mBio. 2022 Jul 12;13(4):e01635-22. doi: 10.1128/mbio.01635-22 (PMC9426505; doi:10.1128/mbio.01635-22)

Figure S1

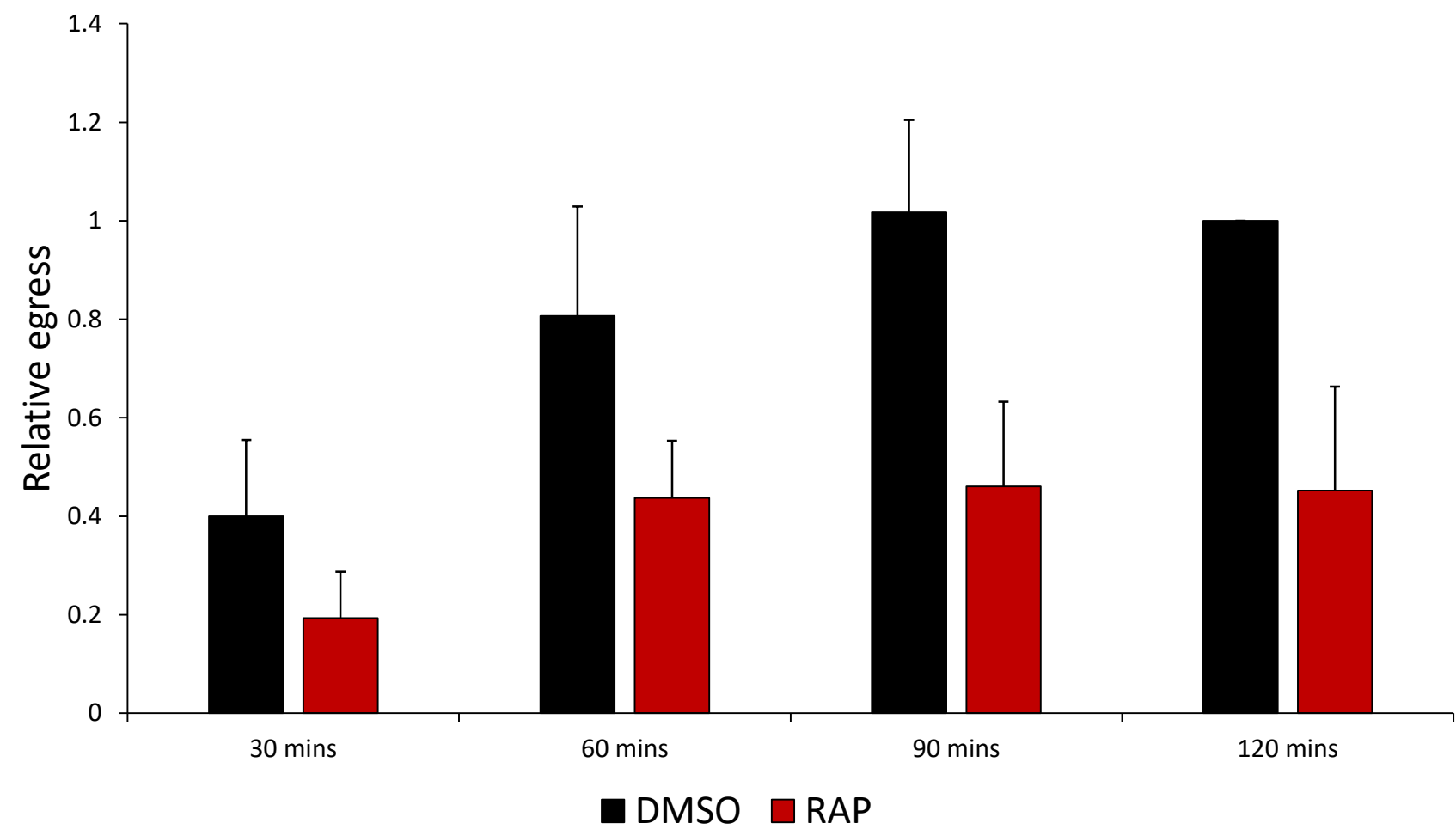

Supplement: FIG S1 [file mbio.01635-22-s0002.pdf]

Figure S2

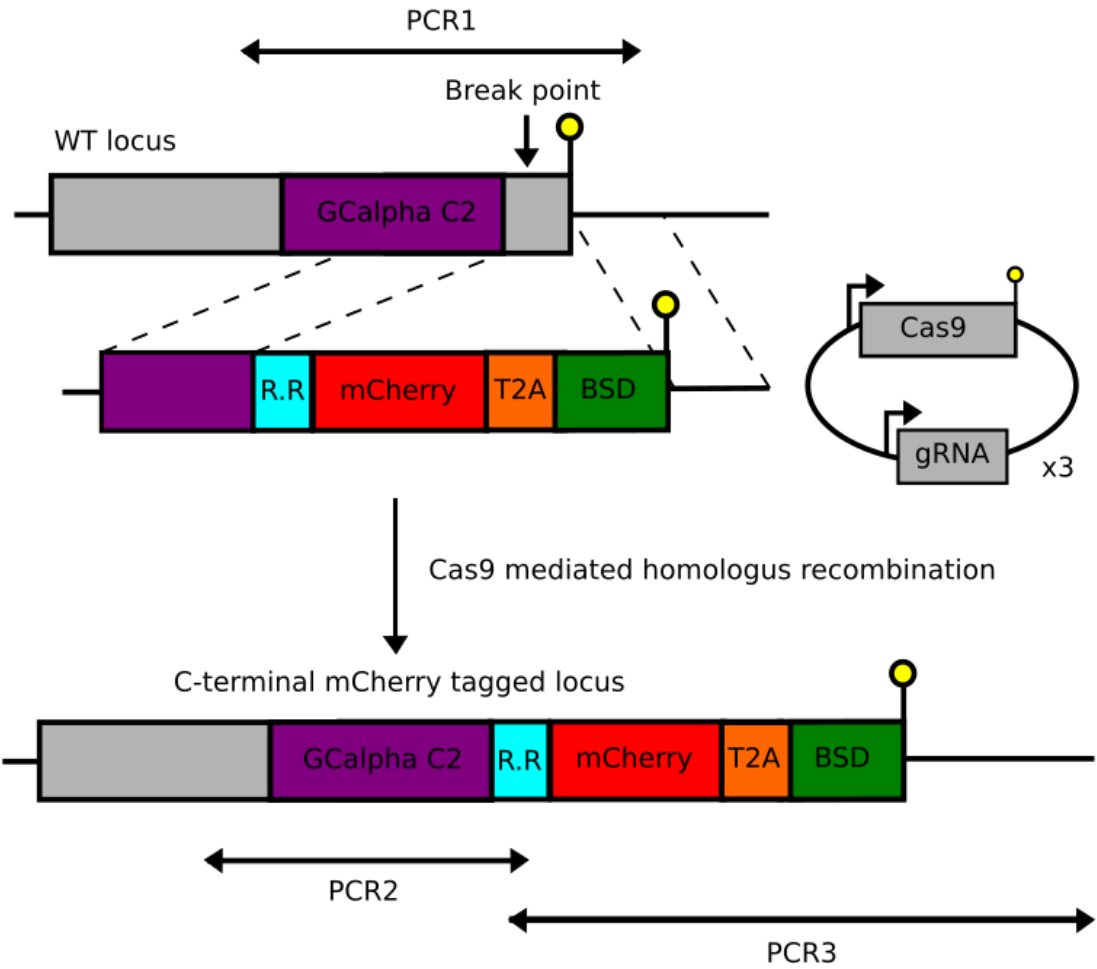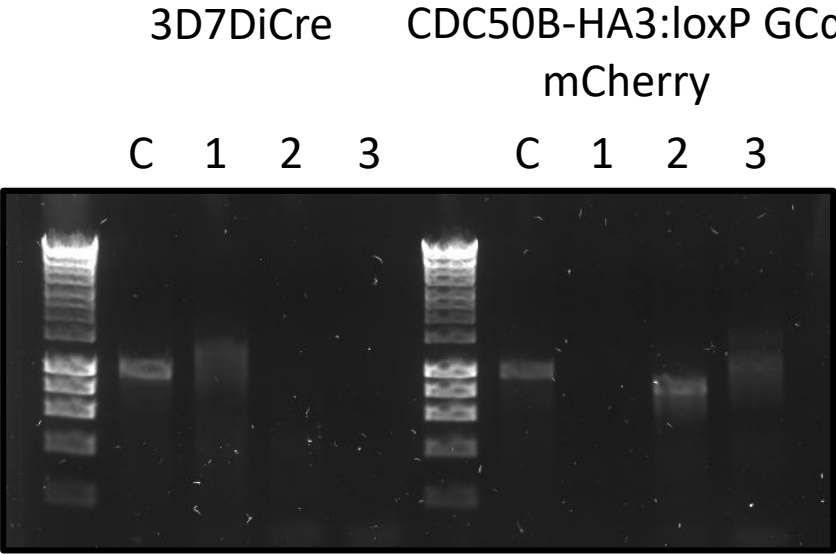

Supplement: FIG S2 [file mbio.01635-22-s0003.pdf]

Figure S3

A)

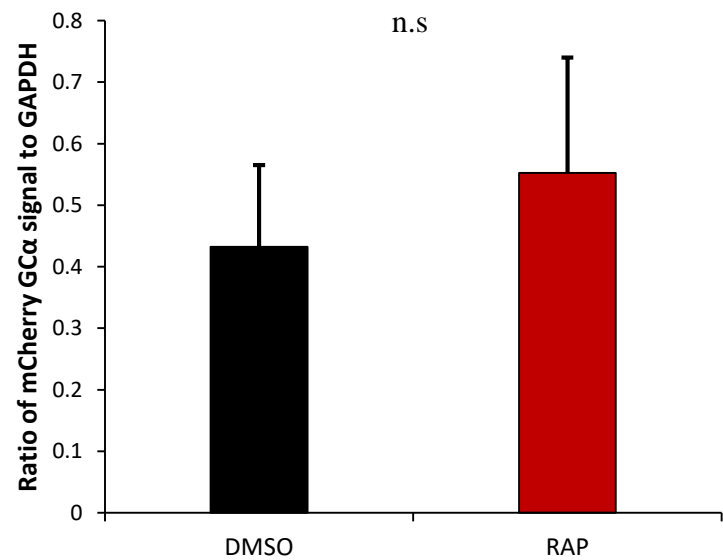

B)

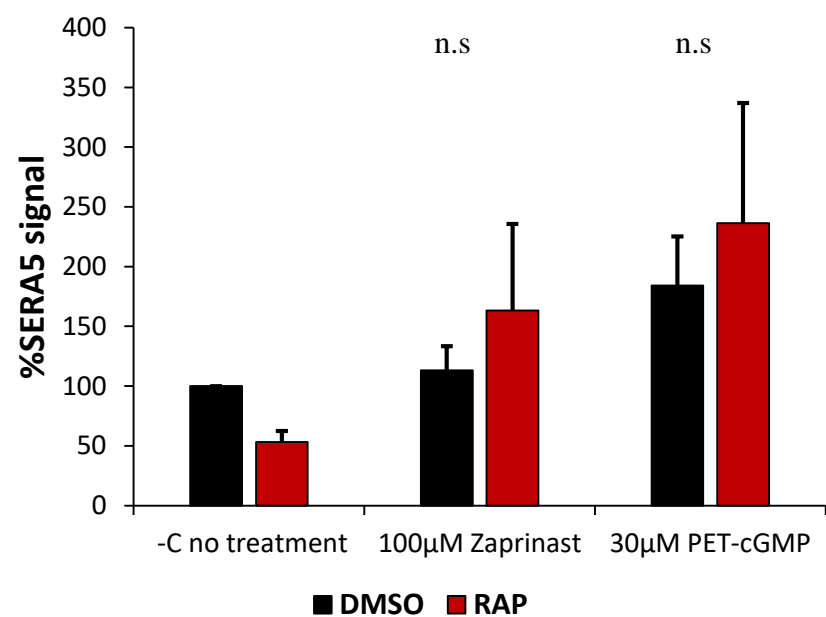

Supplement: FIG S3 [file mbio.01635-22-s0004.pdf]

Figure S4

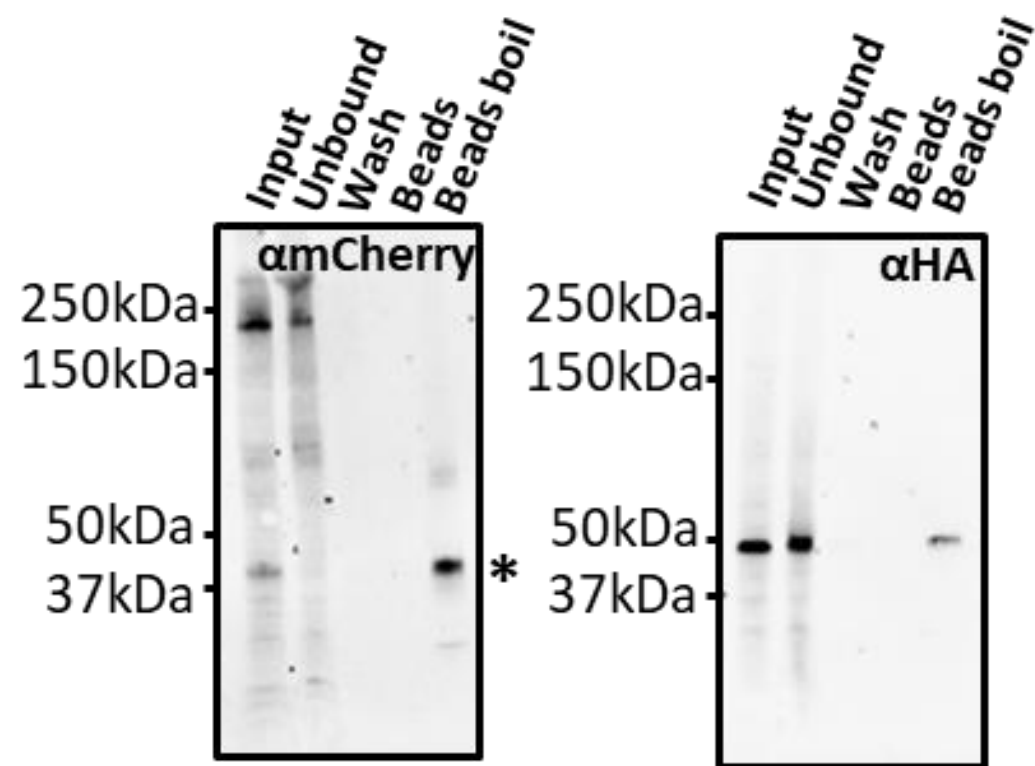

Supplement: FIG S4 [file mbio.01635-22-s0005.pdf]

Figure S5

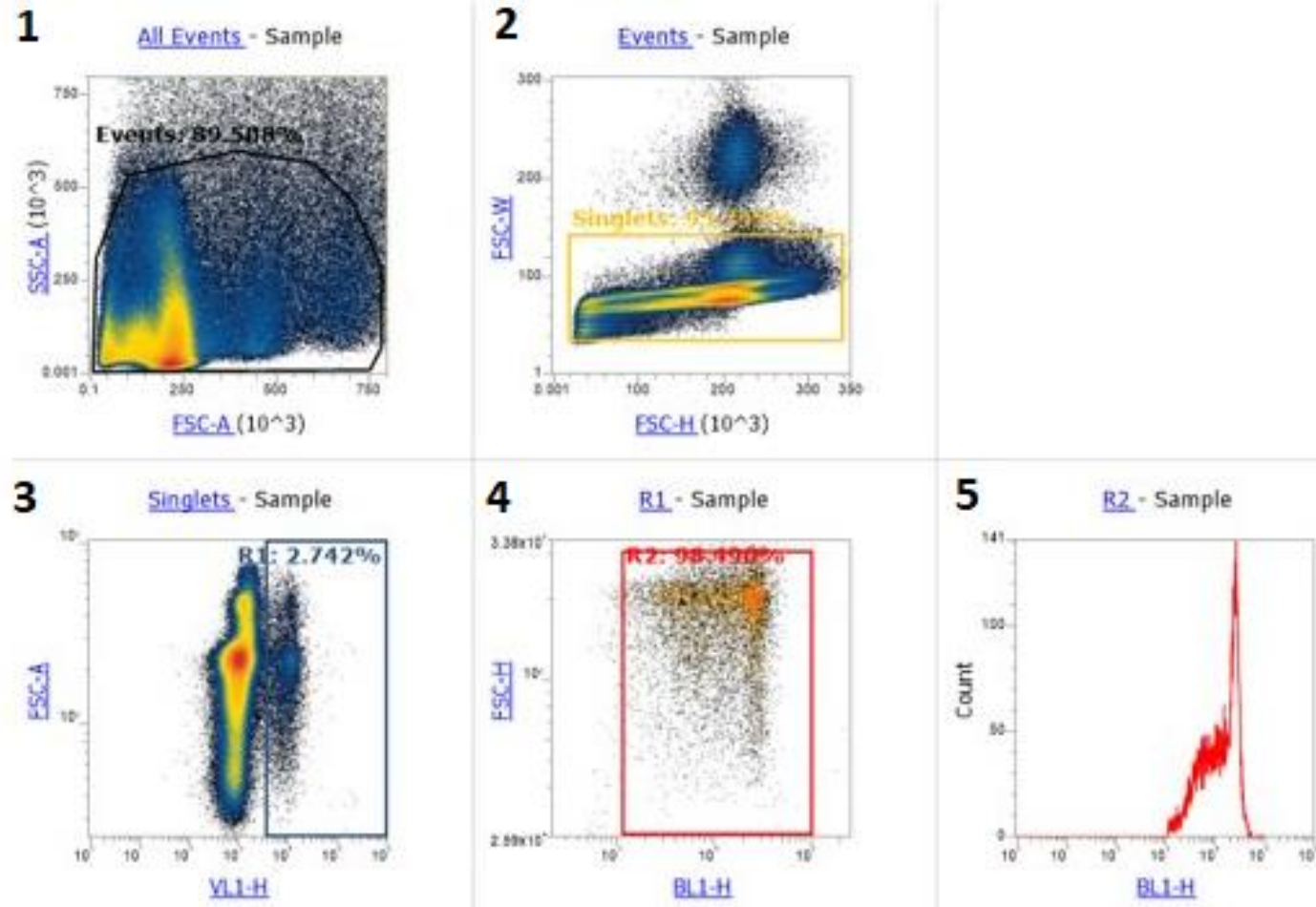

Supplement: FIG S5 [file mbio.01635-22-s0006.pdf]

Figure S6

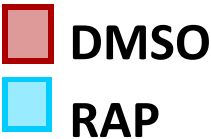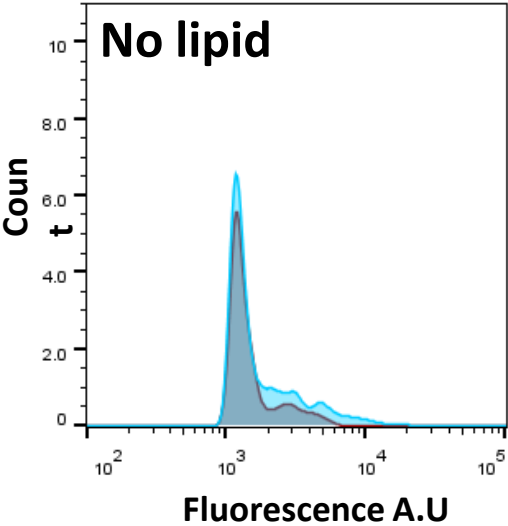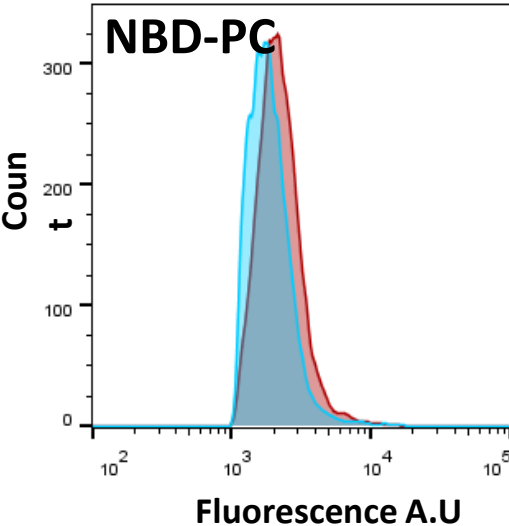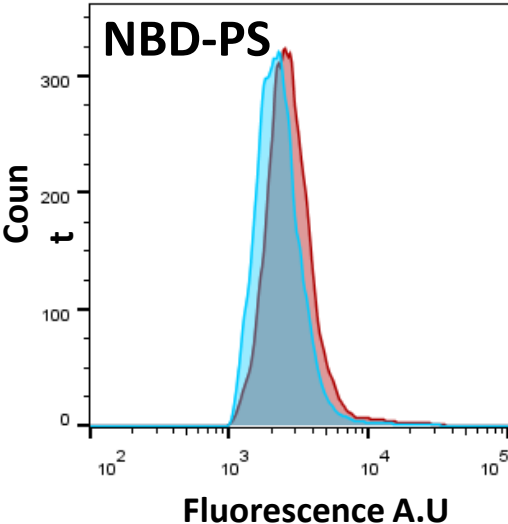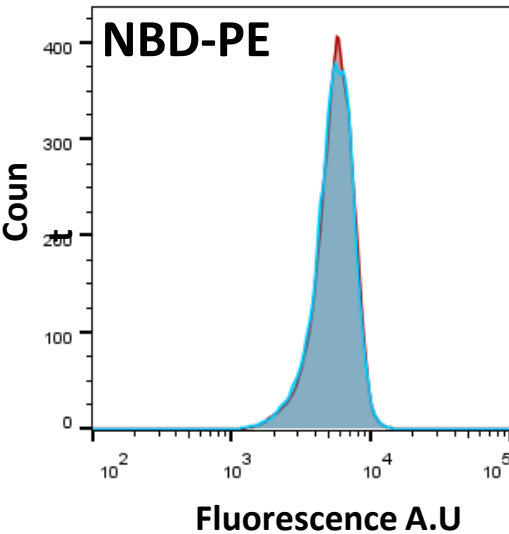

Supplement: FIG S6 [file mbio.01635-22-s0007.pdf]

Figure S7

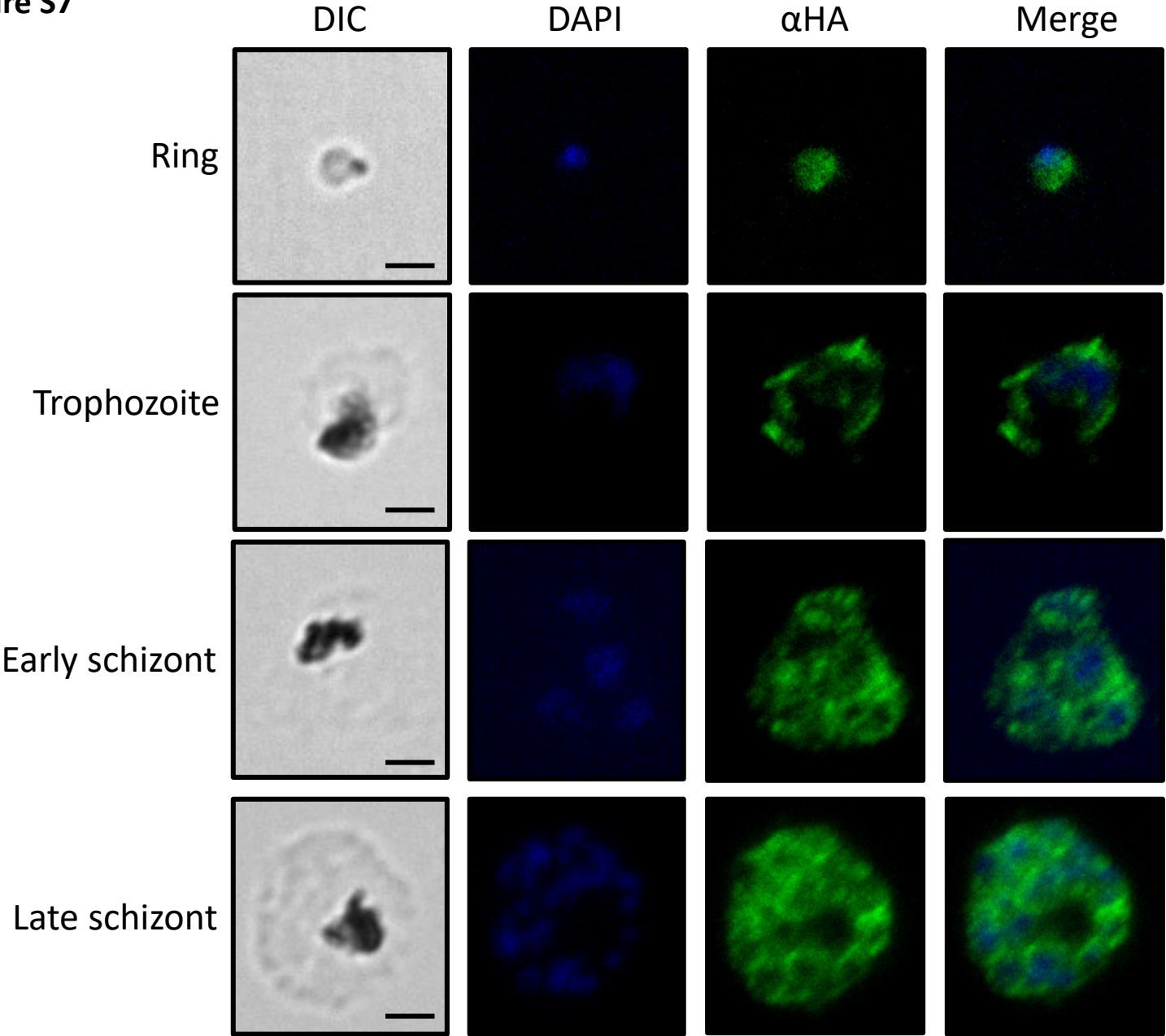

Supplement: FIG S7 [file mbio.01635-22-s0008.pdf]

**Figure S8**

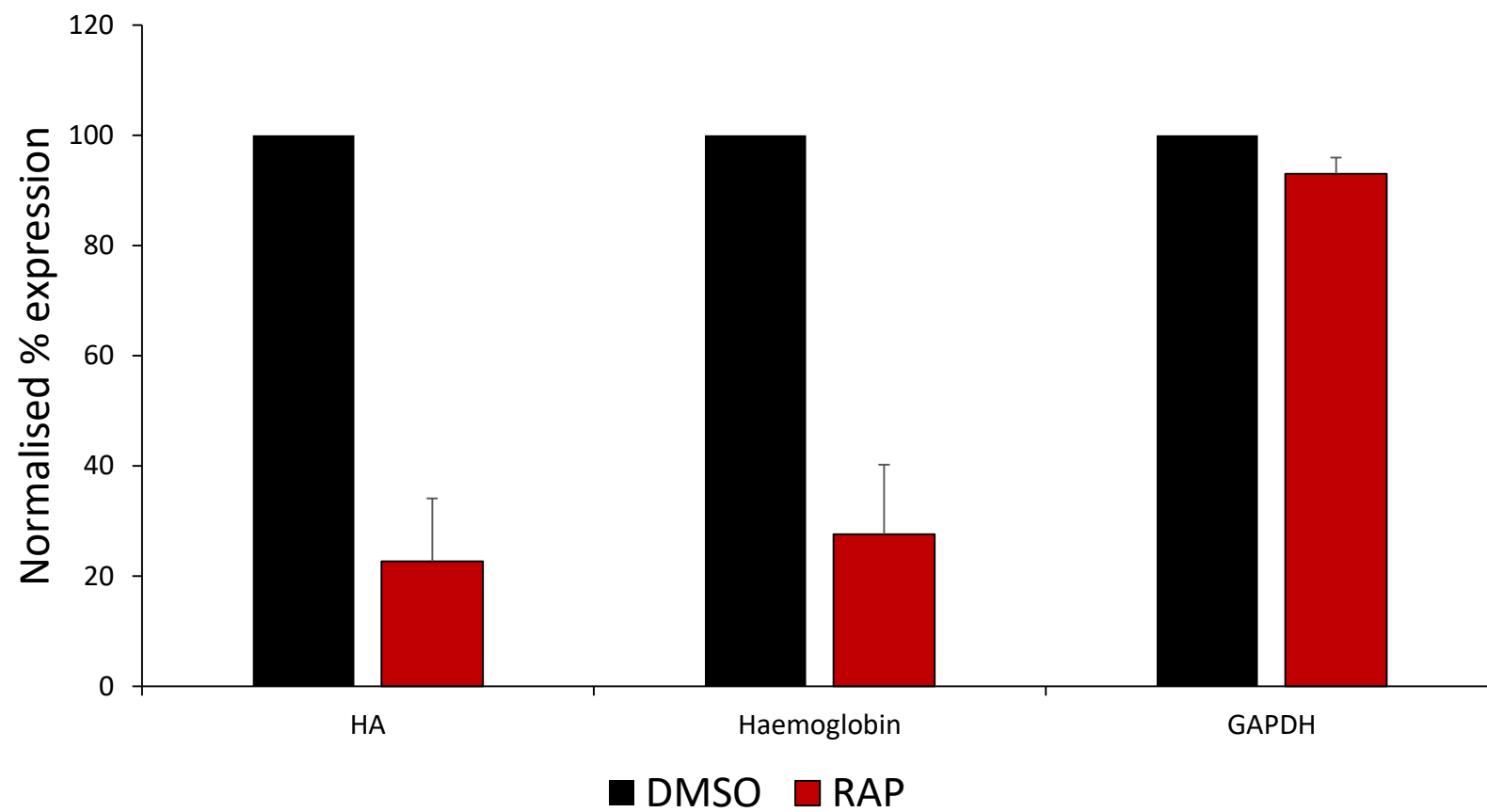

Supplement: FIG S8 [file mbio.01635-22-s0009.pdf]
