# Supplementary material for: CDC50 Orthologues in Plasmodium falciparum Have Distinct Roles in Merozoite Egress and Trophozoite Maturation
Source: mBio. 2022 Jul 12;13(4):e01635-22. doi: 10.1128/mbio.01635-22 (PMC9426505; doi:10.1128/mbio.01635-22)
Supplement: FIG S9 [file mbio.01635-22-s0010.pdf]

Figure S9

A)

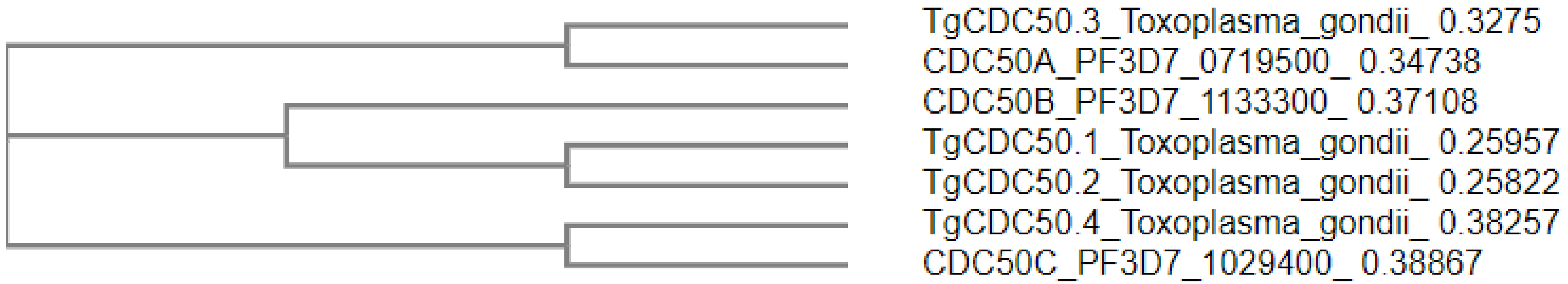
TgCDC50.3_Toxoplasma_gondii_ 0.3275
CDC50A_PF3D7_0719500_ 0.34738
CDC50B_PF3D7_1133300_ 0.37108
TgCDC50.1_Toxoplasma_gondii_ 0.25957
TgCDC50.2_Toxoplasma_gondii_ 0.25822
TgCDC50.4_Toxoplasma_gondii_ 0.38257
CDC50C_PF3D7_1029400_ 0.38867

B)

Alignment of *P. falciparum* CDC50s alongside Cdc50p and LEM3 from *S. cerevisiae and* cdc50a from *H. sapiens* showing the region surrounding the conserved Asn 180 (HsCdc50a) glycosylation site

```
ScCdc50p    LTNFYQNHRRYVQSFDTKQILGEPIKKDDLDTSCSPIRSRED-KIIYPCGLIANSMFNDTFSQVLSGIDDTED-YNLTNKHISWSIDR-HRFKTTKYN  237
ScLem3      LEKFAANHRRYVLSFSEDQIRGEDAS5DATGINCKPLSKNADGKIYYPCGLIANSMFNDTFPLQLTNVGDTSNNYSLTNKGINWESDK-KRYKKTKYN  279
HsCDC50a    LSNFYQNHRRYVKSRDDSQLNGDSSALLNPSKECEPYRRNED-KPIAPCGAIANSMFNDTLELFLIGNDSYPIPIALKKKGIAWWTDKNVKFRNPPGG  220
PfCDC50A    I5NFYQNHKKYLVSKSHNQLMGTVYTKDNEVSQCGPITKNHEGKILHPCGLIARSIFNDTFSVYMDRELHNMIKLDESKEGITWYSDYNKFKNPSDSE  332
PfCDC50B    V5GMQQNHYKFVSGMKKEQLNGNIFLKKEELEECYPLITFSE5KLLHPCGIFPWNVFTDSYIFYDKEPDEVPFPTPLPLK5ITIKYYRQFYKNPSPQN  229
PfCDC50C    VSNFYINFKTFLSDESHSLVNEKIPKKKKNQKCHISDLKPE3KKIFPCGLVSAAIFNDKIALSKNSVNYDIDKFPILH3FLTYMKKHKQFTNYKIWI  335
```

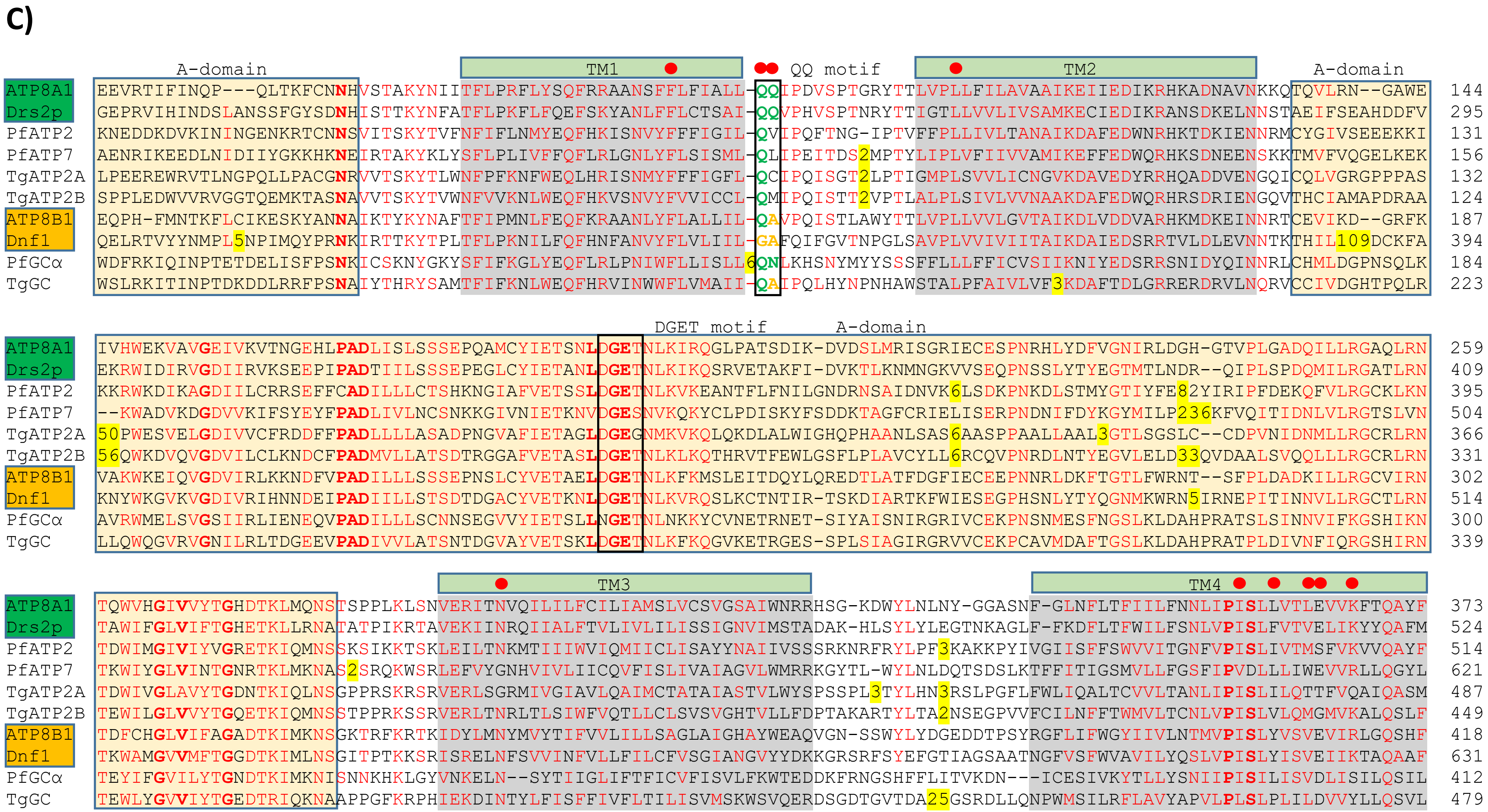
C)
A-domain
TM1
QQ motif
TM2
A-domain
ATP8A1 EEVRTIFINQP---QLTKFCNNHVSTAKYNIITFLPRFLYSQFRRAANSFFLFIALL-QQIPDVSPTGRYTTLVPLLFILAVAAIKEIIEDIKRHKADNAVNKKQTQVLRN--GAWE 144
Drs2p GEPRVIHINDSLANSSFGYSDNHISTTKYNFATFLPKFLFQEFSKYANLFFLCTSAI-QQVPHVSPTNRYTTIGTLLVVLIVSAMKECIEDIKRANSDKELNNSTAEIFSEAHDDFV 295
PfATP2 KNEDDKDVKININGENKRTCNNSVITSKYTVFNFIFLNMYEQFHKISNVYFFFIGIL-QVIPQFTNG-IPTVFFPLLIVLTANAIKDAFEDWNRHKTDKIENNRMCYGIVSEEEKKI 131
PfATP7 AENRIKEEDLNIDIIYGKKHKNEIRTAKYKLYSFLPLIVFFQFLRLGNLYFLSISML-QLIPEITDS2MPTYLIPLVFIIVVAMIKEFFEDWQRHKSDNEENSKKTMVFVQGELKEK 156
TgATP2A LPEEREWRVTLNGPQLLPACGNRVVTSKYTLWNFPFKNFWEQLHRISNMYFFIGFL-QCIPQISGT2LPTIGMPLSVVLICNGVKDAVEDYRRHQADDVENGQICQLVGRGPPPAS 132
TgATP2B SPPLEDWVVRVGGTQEMKTASNAVVTSKYTVWNFVVKNLWEQFHKVSNVYFVVICCL-QMIPQISTT2VPTLALPLSIVLVVNAAKDAFEDWQRHRSDRIENGQVTHCIAMAPDRAA 124
ATP8B1 EQPH-FMNTKFLCIKESKYANNAIKTYKYNAFTFIPMNLFEQFKRAANLYFLALLIL-QAVPQISTLAWYTTLVPLLVVLGVTAIKDLVDDVARHKMDKEINNRTCEVIKD--GRFK 187
Dnf1 QELRTVYYNMPL5NPIMQYPRNKIRTTKYTPLTFLPKNILFQFHNFANVYFLVLIIL-GAFQIFGVTNPGLSAVPLVVIVIITAIKDAIEDSRRTVLDLEVNNTKTHIL109DCKFA 394
PfGCα WDFRKIQINPTETDELISFPSNKICSKNYGKYSFIFKGLYEQFLRLPNIWFLLISLL6QNLKHSNYMYYSSFFLLFFICVSIIKNIYEDSRRSNIDYQINNRLCHMLDGPNSQLK 184
TgGC WSLRKITINPTDKDDLRRFPSNAIYTHRYSAMTFIFKNLWEQFHRVINWWFLVMAII-QAIPQLHYNPNHAWSTALPFAIVLVF3KDAFTDLGRERDRVLNQRVCCIVDGHTPQLR 223
DGET motif
A-domain
ATP8A1 IVHWEKVAVGEIVKVTNGEHLPADLISLSSSEPQAMCYIETSNLDGETNLKIRQGLPATSDIK-DVDSLMRISGRIECESPNRHLYDFVGNIRLDGH-GTVPLGADQILLRGAQLRN 259
Drs2p EKRWIDIRVGDIIRVKSEEPIPADTIILSSSEPEGLCYIETANLDGETNLKIKQSRVETAKFI-DVKTLKNMNGKVVSEQPNSSLYTYEGTMTLNDR--QIPLSPDQMILRGATLRN 409
PfATP2 KKRWKDIKAGDIILCRRSEFFCADILLLCTSHKNGIAFVETSSLDGETNLKVKEANTFLFNILGNDRNSAIDNVK6LSDKPNKDLSTMYGTIYFE82YIRIPFDEKQFVLRGCKLKN 395
PfATP7 --KWADVKDGDVVKIFSYEYFPADLIVLNCSNKKGIVNIETKNVDGESNVKQKYCLPDISKYFSDDKTAGFCRIELISERPNDNIFDYKGYMILP236KFVQITIDNLVLRGTSLVN 504
TgATP2A 50PWESVELGDIVVCFRDDFFPADLLLLASADPNGVAFIETAGLDGEGNMKVKQLQKDLALWIGHQPHAANLSAS6AASPPAALLAAL3GTLSGSLC--CDPVNIDNMLLRGCRLRN 366
TgATP2B 56QWKDVQVGDVILCLKNDCFPADMVLLATSDTRGGAFVETASLDGETNLKLKQTHRVTFEWLGSFLPLAVCYLL6RCQVPNRDLNTYEGVLELD33QVDAALSVQQLLLRGCRLRN 331
ATP8B1 VAKWKEIQVGDVIRLKKNDFVPADILLLSSSEPNSLCYVETAELDGETNLKFKMSLEITDQYLQREDTLATFDGFIECEEPNNRLDKFTGTLFWRNT--SFPLDADKILLRGCVIRN 302
Dnf1 KNYWKGVKVGDIVRIHNNDEIPADIILLSTSDTDGACYVETKNLDGETNLKVRQSLKCTNTIR-TSKDIARTKFWIESEGPHSNLYTYQGNMKWRN5IRNEPITINNVLLRGCTLRN 514
PfGCα AVRWMELSVGSIIRLIENEQVPADILLLSCNNSEGVVYIETSLLNGETNLNKKYCVNETRNET-SIYAISNIRGRIVCEKPNSNMESFNGSLKLDAHPRATSLSINNVIFKGSHIKN 300
TgGC LLQWQGVRVGNILRLTDGEEVPADIVVLATSNTDGVAYVETSKLDGETNLKFKQGVKETRGES-SPLSIAGIRGRVVCEKPCAVMDAFTGSLKLDAHPRATPLDIVNFIQRGSHIRN 339
TM3
TM4
ATP8A1 TQWVHGIVVYTGHDTKLMQNSTSPPLKLSNVERITNVQILILFCILIAMSLVCSVGSAIWNRRHSG-KDWYLNLNY-GGASNF-GLNFLTFIILFNNLIPISLLVTLEVVKFTQAYF 373
Drs2p TAWIFGLVIFTGHETKLLRNATATPIKRTAVEKIINRQIIALFTVLIVLILISSIGNVIMSTADAK-HLSYLYLEGTNKAGLF-FKDFLTFWILFSNLVPISLFVTVELIKYYQAFM 524
PfATP2 TDWIMGIVIYVGRETKIQMNSSKSIKKTSKLEILTNKMTIIIWVIQMIICLISAYYNAIIVSSRKNRFRYLPF3KAKKPYIVGIISFFSWVVITGNFVPISLIVTMSFVKVVQAYF 514
PfATP7 TKWIYGLVINTGNRTKLMKNAS2SRQKWSRLEFVYGNHVIVLIICQVFISLIVAIAGVLWMRRKGYTL-WYLNLDQTSDSLKTFFITIGSMVLLFGSFIPVDLLLIWEVVRLLQGYL 621
TgATP2A TDWIVGLAVYTGDNTKIQLNSGPPRSKRSRVERLSGRMIVGIAVLQAIMCTATAIASTVLWYSPSSPL3TYLHN3RSLPGFLFWLIQALTCVVLTANLIPISLILQTTFVQAIQASM 487
TgATP2B TEWILGLVVYTGQETKIQMNSSTPPRKSSRVERLTNRLTLSIWFVQTLLCLSVSVGHTVLLFDPTAKARTYLTA2NSEGPVVFCILNFFTWMVLTCNLVPISLVLQMGMVKALQSLF 449
ATP8B1 TDFCHGLVIFAGADTKIMKNSGKTRFKRTKIDYLMNYMVYTIFVVLILLSAGLAIGHAYWEAQVGN-SSWYLYDGEDDTPSYRGFLIFWGYIIVLNTMVPISLYVSVEVIRLGQSHF 418
Dnf1 TKWAMGVVMFTGGDTKIMLNSGITPTKKSRISRELNFSVVINFVLLFILCFVSGIANGVYYDKKGRSRFSYEFGTIAGSAATNGFVSFWVAVILYQSLVPISLYISVEIIKTAQAAF 631
PfGCα TEYIFGVILYTGNDTKIMKNISNNKHKLGYVNKELN--SYTIIGLIFTFICVFISVLFKWTEDDKFRNGSHFFLITVKDN---ICESIVKYTLLYSNIIPISILISVDLISILQSIL 412
TgGC TEWLYGVVIYTGEDTRIQKNAAPPGFKRPHIEKDINTYLFISFFIVFLTILISVMSKWSVQERDSGDTGVTDA25GSRDLLQNPWMSILRFLAVYAPVLPLSLPLILDVVYLLQSVL 479

```
                        P-domain          Phosphorylation site                    N-domain
ATP8A1   INWDLDMHYEPTDTAAMARTSNLNEELGQVKYIFSDKTGTLTCNVMQFKKCTIAGVAYGHVPEPEDYGCSPDEWQNSQFGDEK-TFSDSSLLENLQN---NHPTAPIICEFLTMMAV   486
Drs2p    IGSDLDLYYEKTDTPTVVRTSSLVEELGQIEYIFSDKTGTLTRNIMEFKSCSIAGHCYIDKI-PEDKTATVE--DGIEVGYR---KFDDLKKKLNDP---SDEDSPIINDFLTLLAT   632
PfATP2   ISCDKNMIHKV113NAVPRTSSLIEELGQIEYIFSDKTGTLTCNIMEFRKCAINGISYGKGLTEIKRNILKKKNLEIPVEPTMKFKKKTPHVNIIDNDIINH7NHVNLINFFLHLAI   747
PfATP7   INWDNDMYSEKTGRHALSKAGQLLEEMGNVTHIYSDKTGTLTQNVMQLQNIGLG2GTYGFYDFSNIKENITKSRHWGSQDPMDPVLEEYENEENVYSNVN159VQDQIYFLLLVLSL   895
TgATP2A  INSRRVGSSPD272EIVARTSDLNEELGQVSYVFSDKTGTLTENMMEFRKCCIQGIRYGDEVEGEKVRLTQSGGREGLPQEREAPHVKVGDPTLRAALSDPD2MHSHIIDFFLHLAV   874
TgATP2B  IAQDESMFFHP402GAWPRTSDLNEELGQVSYIFSDKTGTMTSNVMEFRKCCVRGLSYGQGLTEVRRQALRRLGLPVPADPLPPPDEPTTPQVQMVDSALRH8MHPYLVDFFLHLAI   972
ATP8B1   INWDLQMYYAEKDTPAKARTTTLNEQLGQIHYIFSDKTGTLTQNIMTFKKCCINGQIYGDHRDASQHNHNK--IEQVDFSWNTYADGKLAFYDHYLIEQIQSGKEPEVRQFFFLLAV   533
Dnf1     IYGDVLLYNAKLDYPCTPKSWNISDDLGQVEYIFSDKTGTLTQNVMEFKKCTINGVSYGRAYTEALAGLRKRQGIDVETEG31VTFVSKEFVRDLKGASGEVQQRCC-EHFMLALAL   776
PfGCα    IENDNHISTFE311WGLCLNSNMHGDLGNVDFIFTDKTGTLTNNNMTFNMCSIAGKTYGSK352KQNFPRPKSFRLNMNSSSVKGRCDFNDTKIYNDLNSQSWRGYYIDEFFKCMTL  1184
TgGC     IEGDIHIRGGG270WAEVHTPALNPNLGQVDFIFTDKTGTITENDMTFSMCSVAGKIYGMA396YDNIDFPTGFRETPMEGQIRRNCDFYDASIFTDLARKDLRSHRINEFIKCMAL  1256

                                                    N-domain                   Adenine-binding
ATP8A1   CHTAVPEREGDKIIYQAASPDEGALVRAAKQLNFVFTGRTPDSV--IIDSLGQEERYELLNVLEFTSARKRMSVIVRTPSGKLRLYCKGADTVIYDRLAETSK-YKEITLKHLEQFA   600
Drs2p    CHTVIPEF2DGSIKYQAASPDEGALVQGGADLGYKFIIRKPNSVTVLLEETGEEKEYQLLNICEFNSTRKRMSAIFRFPDGSIKLFCKGADTVILERLDDEANQYVEATMRHLEDYA   750
PfATP2   NHAVICEK2EGVTTYSSSSPDEEALVNAAKHFDITFLYRREGKY--GISIFGKIYEIDTLATIEFTSKRKMSSVICRIP103IMLFCKGAGSIILKKLAK-RTDVDEITIEHMETYA   962
PfATP7   CHSAMIR35QTQPQYDASSPDELALISTSLYLGCEFVNRPNLTT58MINNKG44LSFEILDVFAFDNVRKRMSLIVKNEKKEIFMLVKGADTSVLKLASKNQENIVDHVEHQLHAFA  1143
TgATP2A  NHSALAEH3TGHLCYSANSPDERAFVCAARHFGVTFRARTPFGV--ELNVLGRTVHVDILASFPFESRRRRSSVLCQIS122VVLLTKGADMVILPRLRRRA51RRKAMETALEAYA  1160
TgATP2B  NHAVVLET2FGMTRYSASSPDEGALVYGARHFGIEFLGQTPSGL--EVSVLGRKLHVRVLASVEFSSKRKRSSMLCEIRY16IVLFTKGADTVILPLLKQ--RREETQMLNTMEEYA  1101
ATP8B1   CHTVMVDRTDGQLNYQAASPDEGALVNAARNFGFAFLARTQNTI--TISELGTERTYNVLAILDFNSDRKRMSIIVRTPEGNIKLYCKGADTVIYERLHR-MNPTKQETQDALDIFA   647
Dnf1     CHSVLVEA4PKKLDLKAQSPDEAALVATARDVGFSFVGKTKKGL--IIEMQGIQKEFEILNILEFNSSRKRMSCIVKIPGL7ALLICKGADSIIYSRLSRQSG5ILEKTALHLEQYA   904
PfGCα    CHAVTP386FDAIKYQSSSLDEECLIYSSSFLGYRLVLRNKNTM--CIEIDGSFNKWTIIGVNEFTNRRGKMSIVVK-PDS4SILYVKGSDSSILSLLNLK140RYRRLEKQLRKFS  1821
TgGC     CNTVVP396VSIIKYQASSPDEECLVSAASHMGYTLVSRTNNYA--ILNINGQERRWQIIGVNEFTQKRGRMSIVVRPQEW3SILYVKGADVAMLDLLSTS231DIALVEQHLRKFS  1994

                                         N-domain                                P-domain  TGD motif
ATP8A1   TEGLRTLCFAVAEISESDFQEWRAVYQRASTSVQNRLLKLEESYELIEKNLQLLGATAIEDKLQDQVPETIETLMKADIKIWILTGDKQETAINIGHSCKLLKKNMGMIVINEGSLD   717
Drs2p    SEGLRTLCLAMRDISEGEYEEWNSIYNEAATTLDNRAEKLDEAANLIEKNLILIGATAIEDKLQDGVPETIHTLQEAGIKIWVLTGDRQETAINIGMSCRLLSEDMNLLIINEETRD   867
PfATP2   DEGLRTLCIAQRELSEESFAEWYHLYKEASLSIKDREEKLESVAEYIENDLILQGITGIEDKLQEGVSSTIEDLRMAGIHIWMLTGDKIETAMNIGIAANLIDNYSEQFIYTEEYIE  1079
PfATP7   TSGLRTLVLGYKYLTENEFSDMYRNHINARKKSE2KEEYLQEFYEEAENNLIIIGCTGIDDKLQDDVPQVIQDLRDAGMTICVLTGDKLETAINIGHSINILNKQTYNAIFTETDPT  1261
TgATP2A  GEGLRTLCIAKRTIDEEEFARWYKEYQKEHTSRT2KEERLYQLAERLEVDLELQGVTAVEDRLQQDVAMTIVKLRQAGIHVWMLTGDKTETALNVGISTFLVARDTRLSRYLWNGRE  1278
TgATP2B  ADGLRTLCIAKREVDTDEFFTWFQAYQQAERATVGRQEQIEAVAERLEVQLELQGITGVEDKLQAGVADTIEKLRAAGIKVWMLTGDKVETAINIGFATSLLTREMTQRTYVWEELD  1218
ATP8B1   NETLRTLCLCYKEIEEKEFTEWNKKFMAASVASTNRDEALDKVYEEIEKDLILLGATAIEDKLQDGVPETISKLAKADIKIWVLTGDKKETAENIGFACELLTEDTTICYEGDINSL   764
Dnf1     TEGLRTLCIAQRELSWSEYEKWNEKYDIAAASLANREDELEVVADSIERELILLGGTAIEDRLQDGVPDCIELLAEAGIKLWVLTGDKVETAINIGFSCNLLNNEMELLVIKTTGDD  1021
PfGCα    VKGLRSMIFAFRYLSEEETIKYKRMYDDACSSIYNKEQRLEKVAEEFERDLIYLGITGVKNGLQEKVPKTIDILNQSGIRIWMLTGDNVEYSLHVSFLCKFLNKHTKIFHAALENSN  1938
TgGC     LQGLRTMALACRYLTQEETETYKRLYTDACASVYCRAERLEEVAEDMERDLEYLGITGVRDKLQEQVPETLQLMMEAGIRVWMVTGDNVEYALHICHSCRLLTSRTRIFHAALEFSG  2111
```

```
                                                          P-domain                Phosphate-binding/ Mg2+ coordination
ATP8A1   GTRETLSRH----------CTTLGDALRKENDFALIIDGKTLKYALTFGVRQYFLDLALSCKAVICCRVSPLQKSEVVEMVKKQVKVVTLAIGDGANDVSMIQTAHVGVGISGNEGL  824
Drs2p    DTERNLLEK-------INALNEHQLSTHDMNTLALVIDGKSLGFALEPELEDYLLTVAKLCKAVICCRVSPLQKALVVKMVKRKSSSLLLAIGDGANDVSMIQAAHVGVGISGMEGM  977
PfATP2   45NFIKNFFCAKNKSGLLLNPDKYNMLINTLNYVLVVDGSVIDLLLSEKMERKFFYLADKCSSVICGRVSPYQKGAIVSSANRLLNKITLAIGDGANDRNMINTANIGIGIRGQEGV 1239
PfATP7   85NSFLESSESFCHNNNEMDKTKGKEKVHYSQFSITITGEALDVIMK-DLKIKFYTLARSASTLIACRVTPKQKSLLVKENSAFNP2TSLAIGDGANDVGMILMANVGVGIAGKEGL 1463
PfATP2A  33KWSGHPRREAEDSRAETGSEGGDQAVREPPEALIVDGEALTFLFE2ERQCAFIRLCCACRAVICCRLAPHQKGAVVSLVKKTTQKVTLAIGDGSNDCNMLLQAHIGVGIRGNEGN 1427
TgATP2B  10EAQEMSILAKHQGKSGRGEAKGSPGRERQAHALVVDGEALQQMLEPDMEQLFVSVCTNCVTVICSRVTPHQKGAVVSLIKRHLQKITLAIGDGANDCNMIQSADIGIGLKGEEGM 1343
ATP8B1   LHARMENQRNRGGVYAKFAPPVQESFFPPGGNRALIITGSWLNEIL37QRQKNFVDLACECSAVICCRVTPKQKAMVVDLVKRYKKAITLAIGDGANDVNMIKTAHIGVGISGQEGM  916
Dnf1     VKEFGSEPSEIVDALLSKYLKEYF16EFPKGNYAIVIDGDALKLALY2DIRRKFLLLCKNCRAVLCCRVSPSQKAAVVKLVKDSLDVMTLAIGDGSNDVAMIQSADVGIGIAGEEGR 1153
PfGCα    AKKLKREGMALYELF-----QLEKEEKKPYENLCLLVNGRNLQTFLN2DLQTHFLNMACTCDVVIACRITAKQKAFLVQLIKNRLY3NTLAIGDGANDIAMIQEANIGVSIMTSDCI 2053
TgGC     RKAKREGVMLYELFR------KARRLKRSDEHICLVVTGPNLRTFLN2DLQTYFLNMACCSDVVVAARVTPSQKAEMVRLVKKRLT3ITMAVGDGGNDVAMLQEAHVGVAIRGKDSA 2225

           P-domain                         TM5                                       TM6
ATP8A1   QAANSSDYSIAQFKYLKNLLMIHGAWNYNRVSKCILYCFYKNIVLYIIEIWFAFVNGFSGQILFERWCIGLYNVMFTAMPPLTLGIFERSCRKENMLKYPELYKTSQNALDFNTKVF  941
Drs2p    DAARSADIAVGQFKFLKKLLLVHGSWSYQRISVAILYSFYKNTALYMTQFWYVFANAFSGQSIMESWTMSFYNLFFTVWPPFVIGVFDQFVSSRLLERYPQLYKLGQKGQFFSVYIF 1094
PfATP2   QAFNSSDYGISQFRFLKNLLLVHGRLSYRRISKLVVYMFYKNMVLIFPLFIFGSISLYSGQKIYFEFLLHLFNVLFTAIPVVIHAVLDQDISLNTAMEKPNLYKLGIHHYYFNIRTF 1356
PfATP7   QAARSSDFTISEFKYLKKLLFVHGRESLRRNSFLVYFCIFRNVSFCLCSMILIFWTGYSAIDAWNPWTKQIINIAFTSLPVIFFVALDKQLPHNILLNNPLLY61YGTHYLFVYLLF 1644
TgATP2A  QAFNCSDFGVTQFRLLLPLLLVHGRWCYRRITTVILYIIYKNFLLVLPLVYFGFLCLFSGQRFYPEVLAQTYNPVLTAMPITLYGVLEQDIDERASLKFPQLYRLGQADSFLNLRTC 1544
TgATP2B  QAFNCSDYGLVQFRFLLPLLLTHGSWNYRRISKLVLYMFYKNLVLVLPMFFFGYISLFSGQKFYFEFLYQMYNVVFTAIPITLYGVFDQDVDKKLALKYPQLYRCGQIDLYLNLRVF 1460
ATP8B1   QAVMSSDYSFAQFRYLQRLLLVHGRWSYIRMCKFLRYFFYKNFAFTLVHFWYSFFNGYSAQTAYEDWFITLYNVLYTSLPVLLMGLLDQDVSDKLSLRFPGLYIVGQRDLLFNYKRF 1033
Dnf1     QAVMCSDYAIGQFRYLARLVLVHGRWSYKRLAEMIPEFFYKNMIFALALFWYGIYNDFDGSYLYEYTYMMFYNLAFTSLPVIFLGILDQDVNDTISLVVPQLYRVGIRKEWNQRKFL 1271
PfGCα    ISAGYSDYCIKKFCYLRKLLFIYGSKHLYTISIILYWNFFKNILLILPIFFYQAYASWSCVKIYPELLYTFFSIFWVFIPIIYYMFLQHNLNYDILYNIPLFYALSRRRYNMNCFKF 2170
TgGC     AA3AYADYSFTEFRFLQRLLFVHGRLSLMRVSTVILWSFFKSLCIGLPTFLFQPQAFWSAVEVYDPLLLMIVDFFWTTLPGIIHGYSDQDLPTHLLPSVPVLYTPGRRRLYFNGFRF 2344

                  TM7                            TM8                            TM9
ATP8A1   WVHCLNGLFHSVILFWFPLKALQYGTAFGNGKTSDYLLLGNFVYTFVVITVCLKAGLETSYWTWF-SHIAIWGSIALWVVFFGIYSSLWPAIPMAPDMSGEAAML--FSSGVFWMGL 1055
Drs2p    WGWIINGFFHSAIVFIGTILIYRYGFALNMHGELADHWSWGVTVYTTSVIIVLGKAALVTNQWTKFTLIAIPGSLLFWLIFFPIYAS--IFPHANISREYYGVVKHTYGSGVFWLTL 1209
PfATP2   ISWVMNSLFHGSVVFLIPLYFLSYYNIPTSDGIPYDIWTVGCATYFLTVLIVNFKILFET---YYLNILPISGIALSIFSFVLLVTAFSFMCVGSIHLLGT----------IVYLVQ 1460
PfATP7   AIWLATVETVIMLHFSTSQILYTHHVIAVNIVVVLMTNTWFFISHVVLWIELIATFFFWF---IISNTKFFLDIIGADELHGTFEKAHSSG--------------------NYYASL 1759
TgATP2A  ISWILLGVVHAAIIFVVGLYAFGYYIIPGPSGQPFDMWMVGTVMMAANCLVANLAILFYS---FSVSAVTWCGIAFSLFSCLVLFFAASTATVGGISTGAIFLLFKASPTICLYVLV 1658
TgATP2B  LKWMLNGVWQAIVIFVVPTFVFGCNAVPTTTGRTMDLWMVGTVMFMMNMIVVNIKVLLET---YYLTTIIWAGFYISLLACLLFVFLFSSWPGFAGSVLGCVFYLFIDAAACAVIAT 1574
ATP8B1   FVSLLHGVLTSMILFFIPLGAYLQTVGQDGE-APSDYQSFAVTIASALVITVNFQIGLDTSYWTFVNAFSIFGSIALYFGIMFDFHSAGIHVLFPSAFQFTGTASNALRQPYIWLTI 1149
Dnf1     WYM-LDGLYQSIICFFFPYLVYHKNMIVTSNGLGLDHRYFVGVYVTTIAVISCNTYVLLHQYRWDW----FSGLFIALSCLVVFAWTGIWSSAIASREFFKAAARIYGAPS-FWAVF 1382
PfGCα    LPWIFEAIFYSMIIYFFAYAALKENSHLNN-GEVITINTFGNICFIGCLLISILRLFLEGSLWSPSILITCFGCFLFVFFPSLLFICFAYLSNEYIREVFRQTFLWAPLYVLLILWF 2286
TgGC     ILWTVEGIIYSFLIFYLLQATWMDGNTFHD-GQVLGFHSYGILLLFGSLLQSNVRIILETSLWTPTFLFTTIVLCTIMFFPTVLLYSVTGWPRRYMELAGRVVFAWPMLYFLIPLWV 2460
```

```
           TM10
ATP8A1     LFIPVASLLLDVVYKVIKRTAFKTLVDEVQELEA 1089
Drs2p      IVLPIFALVRDFLWKYYKRMYEPETYHVIQEMQK 1243
PfATP2     SLRFWLVVILGLFTALLRDYVFKVYKRNFNPEIY 1494
PfATP7     IISLYISILPLIILMTYQFIKKPTMEQIVLEQLK 1793
TgATP2A    ACSLSLGLLWFKRSFRVAFRPELYDVLQRHEYLG 1692
TgATP2B    VAVTSLCLARDWLWKAFRVNCAPQLYHLIQQREY 1608
ATP8B1     ILAVAVCLLPVVAIRFLSMTIWPSESDKIQKHRK 1183
Dnf1       FVAVLFCLLPRFTYDSFQKFFYPTDVEIVREMWQ 1416
PfGCα      STCIISYIFINFTKSILFPNIYNVVNHWLFEQYQ 2320
TgGC       SIGILVQLLLQVFTSSLFPNISGSVKQYLAQKQA 2494
```
